# Colchicine delivered by a novel nanoparticle platform alleviates atherosclerosis by targeted inhibition of NF-κB/NLRP3 pathways in inflammatory endothelial cells

**DOI:** 10.1186/s12951-023-02228-z

**Published:** 2023-11-30

**Authors:** Juan Tang, Tao Li, Xiaojing Xiong, Qiaoyun Yang, Zedazhong Su, Minming Zheng, Qingwei Chen

**Affiliations:** 1 Department of General Practice, The Second Affiliated Hospital of Chongqing Medical University, Chongqing, 400010 China; 2 Department of Ophthalmology, The Second Affiliated Hospital of Chongqing Medical University, Chongqing, 400010 China; 3 Chongqing Key Laboratory of Ultrasound Molecular Imaging, The Second Affiliated Hospital of Chongqing Medical University, Chongqing, 400010 China; 4 Department of Endocrinology, The First People’s Hospital of Ziyang, Sichuan, 641300 China; 5 Department of Ophthalmology, The First People’s Hospital of Ziyang, Sichuan, 641300 China

**Keywords:** Atherosclerosis, Colchicine, Nanoparticles, NF-κB/NLRP3 pathways, Inflammatory endothelial cells

## Abstract

Atherosclerosis, a chronic inflammatory disease characterized by arterial plaque formation, is one of the most prominent causes of cardiovascular diseases. However, the current treatments often do not adequately compromise the chronic inflammation-mediated plaque accumulation and the disease progression. Therefore, a new and effective strategy that blocks atherosclerosis-associated inflammation is urgently needed to further reduce the risk. Colchicine, a potent anti-inflammatory medication, has shown great potential in the treatment of atherosclerosis, but its adverse effects have hampered its clinical application. Herein, we developed a novel delivery nanosystem encapsulated with colchicine (VHPK-PLGA@COL), which exhibited improved biosafety and sustained drug release along with the gradual degradation of PLGA and PEG as confirmed both in vitro and in vivo. Surface modification of the nanoparticles with the VHPK peptide ensured its capability to specifically target inflammatory endothelial cells and alleviate atherosclerotic plaque accumulation. In the ApoE − / − atherosclerotic mouse model, both colchicine and VHPK-PLGA@COL treatment significantly decreased the plaque area and enhanced plaque stability by blocking the NF-κB/NLRP3 pathways, while VHPK-PLGA@COL exhibited enhanced therapeutic effects due to its unique ability to target inflammatory endothelial cells without obvious long-term safety concerns. In summary, VHPK-PLGA@COL has the potential to overcome the key translational barriers of colchicine and open new avenues to repurpose this drug for anti-atherosclerotic therapy.

## Introduction

With the increase of the aging population, cardiovascular disease (CVD) has become a significant public health issue with the highest mortality rate worldwide, accounting for nearly one-in-three deaths [1]. Atherosclerosis (AS), an immune-mediated, chronic and progressive inflammatory process characterized by atherosclerotic plaque formation beneath the endothelium, has been recognized as the major pathological cause of CVD development [2]. Chronic inflammation-mediated endothelial dysfunction is considered the initiative cause of AS [3] that triggers the adhesion and migration of circulating monocytes through vascular walls due to the activation of adhesion molecules and chemokines, thus contributing to atherosclerotic plaque development and progression. Vascular cell adhesion molecule-1 (VCAM-1) (CD106), an immunoglobulin superfamily glycoprotein (100 to 110 kDa), is overexpressed by inflammatory endothelial cells at the sites of atherosclerotic plaque, mediates the adhesion of leukocytes to endothelial cells and facilitates their transmigration to nascent atheromata [4–6]. Inflammation has been implicated in all stages of AS, from monocyte activation to plaque rupture [7]. To date, despite the introduction of the concept of lifestyle measures, noninvasive therapeutic intervention for AS has focused primarily on pharmacologically controlling risk factors such as hypercholesterolemia, hypertension, and hyperglycemia. However, a substantial portion of the population still has CVD [8] even after receiving the optimal treatment, a phenomenon known as residual risk. Hence, it is urgent to identify novel drug targets that can efficiently block AS-associated chronic inflammation, and consequently decrease the persistent residual risk of CVD.

In the landmark clinical trial, the Canakinumab Anti-inflammatory Thrombosis Outcome Study (CANTOS), Ridker and colleagues have demonstrated for the first time that treatment with canakinumab, a fully human monoclonal antibody targeting the proinflammatory mediator interleukin-1β, reduced the risk of cardiovascular events by 15% in patients with stable coronary artery disease (CAD) [9]. However, its wide clinical acceptance was limited due to the observed increase in the number of deaths related to infections and pharmacoeconomic cost [10, 11]. Colchicine (COL), an alkaloid drug, is classically used as an anti-inflammatory drug to treat gout, familial Mediterranean fever, and pericardial disease [12]. Interestingly, multiple large-scale randomized and controlled clinical trials involving patients with stable CAD (the LoDoCo and LoDoCo2 trials) and recent myocardial infarction (COLCOT and COPS trials) have demonstrated that colchicine improved survival by reducing the risk of myocardial infarction, ischemic stroke, and ischemia-driven revascularization by 25–30% [13–15]. However, little is known about the mechanisms by which colchicine exerts its cardiovascular protective effects. Previous studies have shown that colchicine has ability to inactivate the Nod-like receptor family pyrin domain containing protein 3 (NLRP3) inflammasome, thus reducing the release of the proinflammatory cytokines interleukin-1β (IL-1β), interleukin-18 (IL-18), and C-reactive protein (CRP) [16, 17]. In addition, recent studies have found that colchicine exerts cardioprotective effects by modulating the nuclear factor-κB (NF-κB) axis [18, 19]. Frustratingly, the clinical application of colchicine in AS treatment is significantly hampered by its adverse side effects due to its narrow therapeutic dose ranges. An excessive dose of colchicine can cause severe, even lethal, systemic toxicity, including gastrointestinal disturbance and multiorgan failures [20, 21]. Furthermore, long-term treatment with colchicine to alleviate AS and prevent CVD might increase the possibility of adverse effects. Therefore, it is of great importance to optimize the delivery and doses to improve therapeutic efficacy, and concomitantly reduce the adverse effects of colchicine in the treatment of atherosclerotic cardiovascular diseases.

Nanomaterials exhibit tremendous application potential in atherosclerotic plaque treatment [22] due to their structural advantages, such as small size, large surface area, controllable modification, etc., and unique properties applicable for targeted and sustained release of drugs and attenuation of drug adverse effects [23]. Among various nanomaterials, poly(lactic-co-glycolic acid) (PLGA) is an FDA-approved biodegradable and biocompatible copolymer with a long half-life [24, 25]. The use of poly(ethylene glycol) (PEG)-functionalized nanoparticles (NPs) is another effective strategy to increase the biocompatibility and stability, protect the nanosystem from the reticuloendothelial system (RES) and increase the circulation time of the nanoparticles [26]. In addition, the therapeutic efficacy of nanoparticles can be further enhanced after surface modification with the VHPK peptide, which specifically targets VCAM-1 on endothelial cells [4].

In the present study, we developed colchicine-encapsulated nanoparticles (VHPK-PLGA@COL) composed of PLGA and PEG and decorated with the VHPK peptide to specifically target the VCAM-1 molecule that is overexpressed on the surface of inflammatory endothelial cells in AS [4, 27]. We expected that these VHPK-PLGA@COL, compared with free drugs alone, would be dominantly enriched in atherosclerotic plaques and exert enhanced antiatherosclerosis therapeutic efficacy in atheroprone apolipoprotein-E-deficient mice (ApoE − / − mice) with reduced off-target and systemic adverse effects, thereby overcoming the key translational barriers for the clinical application of colchicine in anti-atherosclerotic therapy.

## Materials and methods

### Materials

PLGA-PEG-COOH (PLGA (lactide:glycolide = 50:50, MW: 10000 Da); PEG (MW: 2000 Da)) was purchased from Xi’an Ruixi Biological Technology Co. (Xi’an, China). Dulbecco’s modified Eagle’s medium (DMEM) was purchased from HACAKA (Shanghai, China). Colchicine, polyvinyl alcohol (PVA; MW: 30,000–70,000 Da), the fluorescent dyes 1,1′-dioctadecyl-3,3,3′,3′-tetramethylindocarbocyanine perchlorate (DiI), 1,1′-dioctadecyl-3,3,3′,3′-tetramethylindotricarbocyanine iodide (DiR), 4′,6-diamidino-2-phenylindole (DAPI), N-(3-dimethylaminopropyl)-N’-ethylcarbodii-mide hydrochloride (EDC) and N-hydroxysuccinimide (NHS) were provided by Sigma‒Aldrich Corporation (St Louis, MO, USA). VHPK (VHPKQHRGGSKGC) and FITC-VHPK peptides were purchased from Qiangyao Biological Technology Co. (Suzhou, China). The Cell Counting Kit-8 (CCK-8) assay kit was purchased from Dojindo Laboratories (Mashiki-machi, Tabaru, Japan). Recombinant human tumor necrosis factor α (TNF-α) was purchased from NoVo Protein Scientific, Inc. (Canada). The anti-mouse CD68 antibody, anti-mouse VCAM-1 antibody, anti-mouse MMP-9 antibody, anti-mouse NLRP3 antibody, anti-mouse IL-1β antibody, anti-mouse IL-18 antibody, anti-mouse p65 antibody, and anti-mouse caspase-1 antibody were purchased from Cell Signaling Technology (MA, USA). Enzyme-linked immunosorbent assay (ELISA) kits were purchased from Boster Biological Technology Co. Ltd. (Wuhan, China). Oil red O (ORO) and hematoxylin and eosin (H&E) were purchased from Servicebio (Wuhan, China). Human umbilical vein endothelial cells (HUVECs) were purchased from Science Cell (Carlsbad, CA).

### Preparation of VHPK-PLGA@COL and confirmation of VHPK peptide binding

#### Preparation of VHPK-PLGA@COL

PLGA@COL was prepared using a modified double-emulsion method (W/O/W method) [28, 29]. According to this method, 50 mg of PLGA-PEG-COOH and 25 mg of colchicine were fully dissolved in 2 mL of chloroform (CHCl_3_) as the oil phase. Then, 200 μL of double-distilled water was added to serve as the inner aqueous phase, and the mixture was emulsified with a sonicator (Sonics & Materials Inc., Newtown, Connecticut, USA) to obtain a primary emulsion. Subsequently, 5 mL of 4% PVA solution was added for the second sonication cycle to form a W/O/W double emulsion. Ten milliliters of 2% isopropanol solution was then added, and the solution was magnetically stirred for 4 h until the organic solvents had evaporated completely and the surfaces of the NPs solidified. Finally, PLGA@COL was purified by centrifugation (10,000 rpm, 7 min).

VHPK-PLGA@COL was fabricated by the carbodiimide method [30]. Briefly, excess EDC and NHS at a molar ratio of 2:1 were added to 0.1 M MES buffer solution (pH = 5.2) to activate the carboxyl group of PLGA@COL, and the mixture was allowed to react on a shaker for 2 h. The unreacted EDC and NHS were removed by centrifugal washing. Then, the activated PLGA@COL and 5 mg of VHPK peptide were dispersed in 0.1 M MES buffer solution (pH = 8) and reacted in a shaker for 12 h. Finally, VHPK-PLGA@COL was rinsed with double-distilled water three times to remove the unreacted materials.

DiI- or DiR-labeled PLGA@COL or VHPK-PLGA@COL was added to an appropriate amount of DiI or DiR when dissolving PLGA-PEG-COOH. We fabricated FITC-labeled NPs using a similar method except we replaced the VHPK peptide with FITC-VHPK peptide. VHPK-PLGA was prepared using the same procedures described above without the addition of colchicine in the first step as a blank control.

#### Confirmation of VHPK peptide binding

To confirm the conjugation of the VHPK peptide to PLGA@COL, VHPK-PLGA@COL was visualized by observing the colocalization of DiI-labeled PLGA@COL (red) and FITC-labeled VHPK peptide (green) using confocal laser scanning microscopy (CLSM; A1R, Nikon, Tokyo, Japan). The carrier rate of the VHPK peptide was analyzed by flow cytometry (FCM; FACS Vantage SE, Becton Dickinson, San Jose, CA, USA). To further evaluate the density of VHPK peptide on VHPK-PLGA@COL, the concentration of VHPK peptide in the supernatant was determined using high-performance liquid chromatography (HPLC, Kromasil 100-5C18: 5 µm, 4.6 mm × 250 mm; Temperature: 25 ℃; Mobile phase: 0.1% Trifluoroacetic Acid in Acetonitrile and 0.1% Trifluoroacetic Acid in water; Flow Rate: 1.0 ml/min; Run Time: 20 min; Wavelength: 220 nm). The VHPK peptide binding capacity (BC) and binding efficiency (BE) were calculated as follows:

$$BC\,(\% )\, = \,\frac{mass\,of\,VHPK\,peptide\,binding\,on\,nanoparticles}{{mass\,of\,nanoparticles}}\, \times \,100\%$$

$$BE\,(\% )\, = \,\frac{mass\,of\,VHPK\,peptide\,binding\,on\,nanoparticles}{{mass\,of\,VHPK\,peptide\,used}}\, \times \,100\%$$

The mass of VHPK peptide on nanoparticles = mass of VHPK peptide used – mass of VHPK peptide in the supernatant.

### Characterization of VHPK-PLGA@COL

The morphology and structure of VHPK-PLGA@COL were observed using scanning electron microscopy (SEM; Hitachi S-3400N, Hitachi, Ltd., Tokyo, Japan) and transmission electron microscopy (TEM; Hitachi H-7600, Hitachi, Ltd., Tokyo, Japan). The size, polydispersity indexes (PDIs), and zeta potentials of VHPK-PLGA and VHPK-PLGA@COL were measured by a dynamic light scattering detector (DLS, Malvern Instruments, Malvern, UK). To test the stability of VHPK-PLGA@COL, the size and PDI were monitored using DLS in phosphate-buffered saline (PBS) containing 10% fetal bovine serum (FBS) for 7 d.

The standard curve of colchicine dissolved in double-distilled water was established by measuring the absorbance of solutions with different concentrations with a UV‒vis–NIR spectrophotometer at a wavelength of 353 nm. The concentration of colchicine was calculated based on the corresponding absorbance of the UV spectrum at 353 nm. The drug loading capacity (LC) and encapsulation efficiency (EE) were calculated as follows:

$$LC\,(\% )\, = \,\frac{mass\,of\,COL\,encapsulated\,on\,nanoparticles}{{mass\,of\,nanoparticles}}\, \times \,100\%$$

$$EE\,(\% )\, = \,\frac{mass\,of\,COL\,encapsulated\,on\,nanoparticles}{{mass\,of\,COL\,used}}\, \times \,100\%$$

The mass of COL encapsulated in nanoparticles = mass of COL used—mass of COL in the supernatant.

### Drug release analysis

#### In vitro drug release analysis

The cumulative release of colchicine from VHPK-PLGA@COL was investigated by adding the nanoparticles into a dialysis bag (MWCO 3500 Da) and immersing it in 50 mL of PBS (pH 7.4) at 37 °C or 4 °C with shaking at 100 rpm. At specific time intervals (0, 0.5, 1, 1.5, 2, 2.5, 3, 6, 10, 16, 20, 24, 30, 39, and 48 h), 1 mL of sample was withdrawn from the buffer solution and replaced with an equal volume of fresh PBS. As a control, an equal amount of free colchicine was added to a dialysis bag (MWCO 3500 Da) at 37 °C with shaking at 100 rpm. The cumulative release of colchicine was calculated according to the standard curve.

#### In vivo drug release analysis

Male SD rats were purchased from the Animal Center of Chongqing Medical University. Rats were randomly divided into two groups (n = 3 for each group). Colchicine and VHPK-PLGA@COL were intravenously administered via the tail vein at an equivalent dose of 0.1 mg/kg colchicine. Blood samples (200 µL) were drawn from the carotid vein at the preset time points, e.g., 0.25, 0.5, 0.75, 1, 2, 4, 8, 12, 24, and 48 h, after intravenous administration, and centrifuged immediately at 3000 rpm for 5 min to obtain plasma. The plasma was placed in a 3 KDa centrifugal filter and centrifuged for 10 min at 10,000 rpm to separate free colchicine from plasma. The ultrafiltrate (50 µL) was placed in a glass tube with 50 µL of the internal standard (20 ng/ml tegafur), and 2 ml of *n*-hexane:dichloromethane:isopropanol (300:150:15, v/v/v) was added. The mixture was vortexed for 3 min and centrifuged at 3500 rpm for 10 min. Then, the upper organic layer was decanted into another tube and evaporated to dryness at 40 ℃ under a gentle stream of nitrogen. The residue was reconstituted in 50 µL of mobile phase and then was injected into a liquid chromatography–tandem mass spectrometer (LC–MS/MS) to measure the colchicine concentration. The LC–MS/MS system consisted of an HPLC (Agilent Technologies, Palo Alto, CA, USA) and a mass spectrometer (MS, Applied Biosystems Sciex, Ontario, Canada) using electrospray ionization (ESI). Chromatography was performed on a Zorbax Extend C18 column (5 µm, 150 mm × 4.6 mm i.d. Agilent Technologies) maintained at 40 ℃ with a mobile phase of formic acid:10 mM ammonium acetate:methanol (1:49:75, v/v/v) at a flow rate of 1.1 ml/min. For quantitative analysis, the MS was run in multiple reaction monitoring (MRM) mode. The MRM transitions for colchicine were 400.1 → 358.3 (m/z) and for tegafur were 200.5 → 130.9 (m/z).

### Cell culture and establishment of the inflammatory cell model

HUVECs were cultured in DMEM supplemented with 10% FBS and 1% penicillin/streptomycin and incubated at 37 °C in a 5% CO_2_ atmosphere. Cultured cells in the logarithmic growth phase were used for cell experiments. Inflammatory endothelial cells were induced by incubation with 20 ng/mL TNF-α [4] at 37 °C for 24 h.

### Establishment of the mouse model of atherosclerosis

Six-week-old homozygous male apolipoprotein E knockout C57BL/6 mice (ApoE − / − mice) were purchased from Beijing Huafukang Biotechnology Co., Ltd. (License SCXK 2019e0008), quarantined, and acclimatized for one week before the experiments. All mice were subjected to a 12 h light/dark cycle under specific pathogen-free conditions at 27 °C and properly handled in accordance with the guidelines of the Institutional Animal Care and Use Committee (IACUC) of Chongqing Medical University. All animal experiments were approved by the Animal Ethics Committee of Chongqing Medical University. ApoE − / − mice were fed a high-cholesterol diet (HCD; 40% fat, 40% carbohydrate, and 20% protein) with a high level of cholesterol (D12108C-high-fat rodent diet with 1.25% cholesterol, FBSH, Shanghai, China) for 10 weeks [4, 31] to induce atherosclerotic plaque formation in the mouse aortic region and establish an atherosclerotic mouse model. As the normal group, six-week-old ApoE − / − mice were fed a normal chow diet (NCD; 10% fat, 70% carbohydrate, and 20% protein).

### In vitro cytotoxicity and blood compatibility tests of VHPK-PLGA@COL

#### Cytotoxicity test

The toxicity of colchicine and VHPK-PLGA@COL was detected by CCK-8 assays, live/dead cell staining, and FCM. First, HUVECs were seeded in a 96-well plate at a density of 1 × 10^4^ cells per well and incubated for 24 h. The cells were treated with colchicine or VHPK-PLGA@COL at different concentrations (0.1, 0.2, 0.4, 0.8, 1, 30 and 50 μg/mL) for 24 h and 48 h, respectively. Cell viability was measured using a CCK-8 assay. In addition, HUVECs (5 × 10^5^) were seeded in laser confocal cell culture dishes and cultured for 24 h, followed by treatment with 0.8 µg/mL colchicine and VHPK- PLGA@COL for 24 h. Then, the cells were stained with M5 HiPer Calcein AM/PI and observed by CLSM (Beijing, China). Meanwhile, the cell survival rate following drug treatment for 24 h was determined by FCM.

#### Blood compatibility test

For the hemolysis evaluation, fresh blood was collected from anesthetized C57BL/6 mice with anticoagulant containing ethylenediaminetetraacetic acid (EDTA), and the whole blood was diluted with saline to obtain diluted whole blood. Twenty microliters of the diluted whole blood was added to 1 mL of VHPK-PLGA@COL solution at various concentrations (0.4, 0.8, 2, 3, 4, and 5 µg/mL) and incubated at 37 °C for 1 h. Red blood cells in saline were used as negative controls, and those in double-distilled water were regarded as positive controls. Subsequently, the supernatant of each sample was collected by centrifugation (3000 rpm, 5 min), and the absorbance at 540 nm was measured using a microplate reader.

### Analysis of the targeting ability of VHPK-PLGA@COL

#### Targeting ability analysis in vitro

The ability of VHPK-PLGA@COL to target HUVECs was evaluated using both CLSM and FCM. The inactivated and activated cells (induced by 20 ng/mL TNF-α) were seeded into laser confocal cell culture dishes and cultured for 24 h. Then, the original medium was replaced with fresh medium containing 0.4 µg/mL DiI-labeled VHPK-PLGA@COL or PLGA@COL and incubated for another 2 h. After washing three times with PBS, the cells were fixed with 4% paraformaldehyde for 15 min and stained with DAPI solution for another 10 min, followed by washing twice with PBS. Cell images were captured by CLSM. For the blocking experiment, activated HUVECs were cultured with medium containing free VHPK peptide solution overnight, followed by incubation with medium containing 0.4 µg/mL DiI-labeled VHPK-PLGA@COL for 2 h. Cell images were captured by CLSM. For dynamic uptake analysis, activated HUVECs were incubated with 0.4 µg/mL DiI-labeled VHPK-PLGA@COL or PLGA@COL for various periods (0, 0.5, 2, and 4 h), and the uptake of VHPK-PLGA@COL or PLGA@COL by HUVECs was quantitatively determined and analyzed by FCM.

#### In vivo targeting analysis

To determine the biodistribution and targeting capability of VHPK-PLGA@COL in vivo, equal volumes of DiR-labeled VHPK-PLGA@COL and PLGA@COL or saline as the control group were intravenously administered to ApoE − / − atherosclerotic mouse models (3 mice/group). After 24 h, the aortas from the aortic root to the bifurcation of the iliac artery, hearts, livers, spleens, lungs, and kidneys were dissected from different groups of mice and imaged by an in vivo imaging system (IVIS, Perkin Elmer, U.K.). In addition, sections of the isolated aortic sinuses were directly immunofluorescence staining of VCAM-1 (green) followed by staining with DAPI solution to determine the distribution of VHPK-PLGA@COL and PLGA@COL by CLSM.

### Animal experiments

#### Animal experimental protocol

**Fig. 1:**
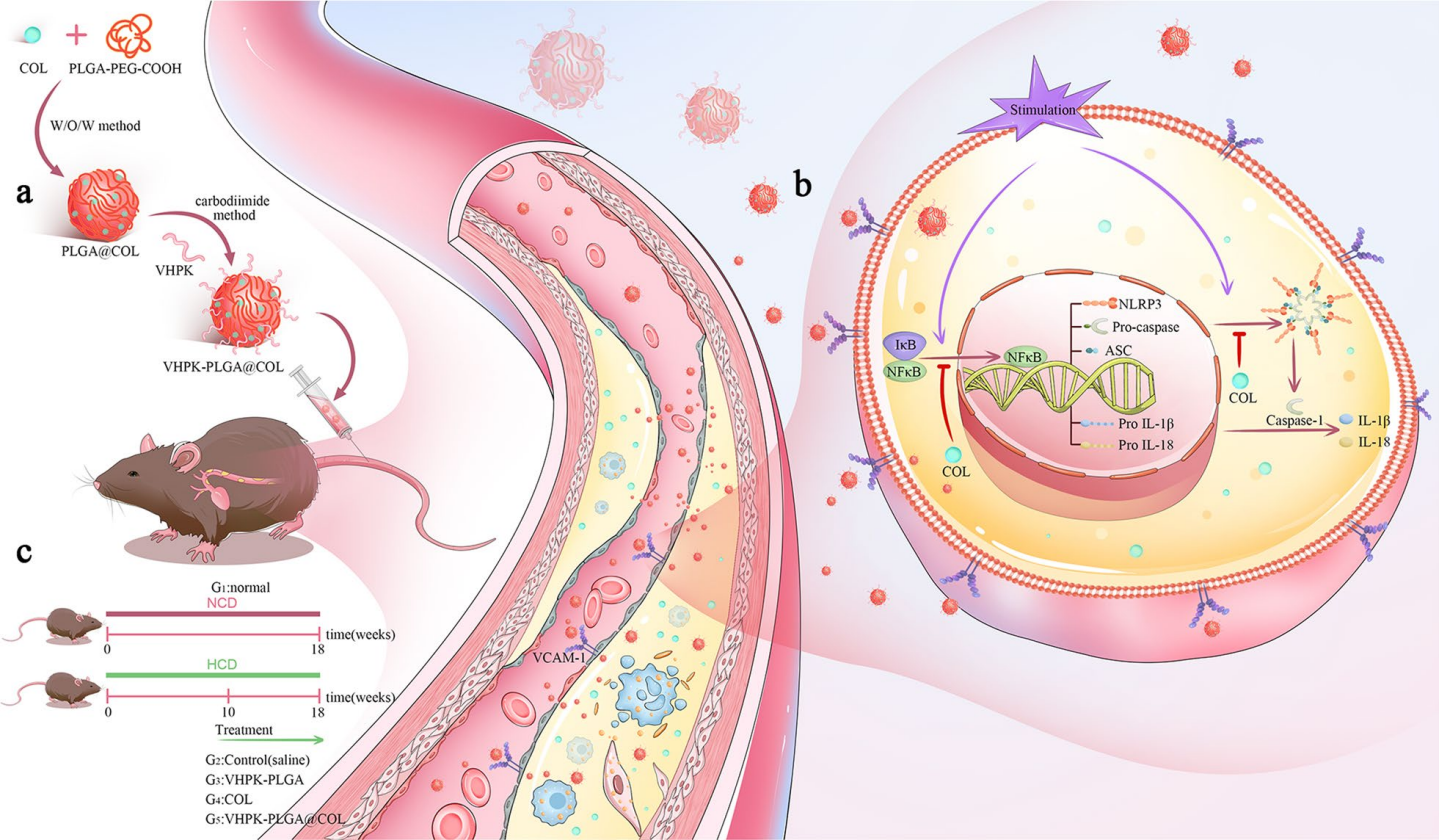
Schematic illustration of the preparation of VHPK-PLGA@COL and the therapeutic paradigm and potential mechanisms. **a** After encapsulating COL in the nanoparticles, the VHPK peptide was subsequently added to target AS, forming VHPK-PLGA@COL. **b** These nanoparticles could accumulate in inflamed endothelial cells that overexpressed VCAM-1 and restrict the progression of AS by inhibiting NF-κB/NLRP3 pathways and reducing the secretion of proinflammatory cytokines such as IL-1β and IL-18. **c** In our study, six-week-old male ApoE − / − mice were divided into 5 groups (G1-G5). In G1, the normal group, mice were fed a normal chow diet (NCD). In G2 to G5, mice were fed a high-cholesterol diet (HCD) for 10 weeks followed by treatment with saline, VHPK-PLGA, COL, or VHPK-PLGA@COL, respectively, for 8 weeks

As illustrated in the scheme (Fig. 1c), six-week-old male ApoE − / − mice were randomly and investigator-blindly divided into 5 groups (G1-G5: normal, control, VHPK-PLGA, colchicine, VHPK-PLGA@COL; 5 mice/group). Mice in G1, the normal group, were fed a normal chow diet (NCD) throughout the experimental period. All mice from G2 to G5 were first fed a high-cholesterol diet (HCD) for 10 weeks to establish the atherosclerotic model followed by treatment with saline as the control (G2), VHPK-PLGA (G3), colchicine (G4), and VHPK-PLGA@COL (G5), respectively.

The indicated formulations were administered intravenously via the tail vein every two days for 8 weeks at a dosage of 0.1 mg/kg colchicine and an equivalent amount of saline. The colchicine dosage used was based on previously published studies [32, 33]. Upon termination of the study, the body weights of mice were recorded, and the animals were sacrificed under anesthesia. Blood was collected in EDTA spray-coated tubes and centrifuged at 3000 rpm for 5 min to collect plasma. The aortas from the heart to the iliac bifurcation and organs were carefully dissected and fixed with 4% paraformaldehyde solution.

#### In vivo biosafety evaluation of VHPK-PLGA@COL

Hematological parameters, including red blood cells (RBCs), platelets (PLTs), white blood cells (WBCs), and hemoglobin (HGB) content, in blood samples collected from different groups of mice were analyzed, respectively. Meanwhile, biochemical parameters in the corresponding plasma samples, including alanine aminotransferase (ALT), aspartate aminotransferase (AST), creatinine (CRE), blood urea nitrogen (BUN), total cholesterol (TC), triglyceride (TG), low-density lipoprotein cholesterol (LDL-C), and high-density lipoprotein cholesterol (HDL-C), were also evaluated accordingly. In addition, the major organs of mice, including heart, liver, spleen, lung, and kidney, were harvested and fixed with 4% paraformaldehyde for H&E staining.

#### Evaluation of the in vivo anti-atherosclerotic effect of VHPK-PLGA@COL

##### Quantitative analysis of the atherosclerotic plaques

Digital images of the aortic arch were obtained after the aortas were dissected from the mice. The plaque shape, size, and distribution throughout the entire aorta were observed intuitively with gross pathological specimens. The whole aorta was longitudinally opened and stained with ORO, and photos of the stained aortas were quantitatively analyzed by ImageJ to evaluate the therapeutic efficacy of the different formulations.

##### Assessment of the stability of atherosclerotic plaques

To assess stability of the atherosclerotic plaques, sections of the aortic sinus were stained with H&E and ORO and incubated with antibodies, including anti-CD68 and anti-matrix metalloproteinase-9 (MMP-9), respectively, for immunofluorescence analysis. Then, the sections were visualized using CLSM. Furthermore, quantitative analysis of atherosclerotic plaques or the positive area was determined by ImageJ.

#### Quantification of inflammatory cytokines in plasma

The levels of TNF-α, IL-1β, IL-18, and CRP in the plasma of mice were analyzed and quantified using commercial ELISA kits according to the manufacturer’s instructions.

#### Western blot

Total proteins were extracted from entire aortas dissected from different groups of mice. The protein concentration was determined using BCA assay. Equivalent amounts of denatured protein samples were separated by 12% SDS‒PAGE, transferred to PVDF membranes (Bio-Rad Laboratories, Hercules, CA, USA), and blocked with 5% bovine serum albumin (BSA) for 1 h at room temperature. The membranes were then incubated overnight at 4 °C with primary antibodies against NF-κB p65, NLRP3, caspase-1, IL-1β, IL-18, α-Tubulin, and GAPDH, followed by incubation with HRP-conjugated secondary antibodies for 1 h at room temperature. The blot signals were visualized by enhanced chemiluminescent (ECL) reagents and detected through a Bio-Rad imaging system (Bio-Rad, USA). The expression level of the target proteins was semi-quantified by measuring the relative gray value of each target protein band with the corresponding GAPDH or α-Tubulin as internal control for the normalization of data.

#### Statistical analysis

All statistical analysis were performed using GraphPad Prism (version 8.02). One-way analysis of variance (ANOVA) was performed for multiple comparisons. All data are displayed as the mean ± SD and *P* values < 0.05 were considered statistically significant.

## Results

### Preparation of VHPK-PLGA@COL and confirmation of VHPK binding

**Fig. 2:**
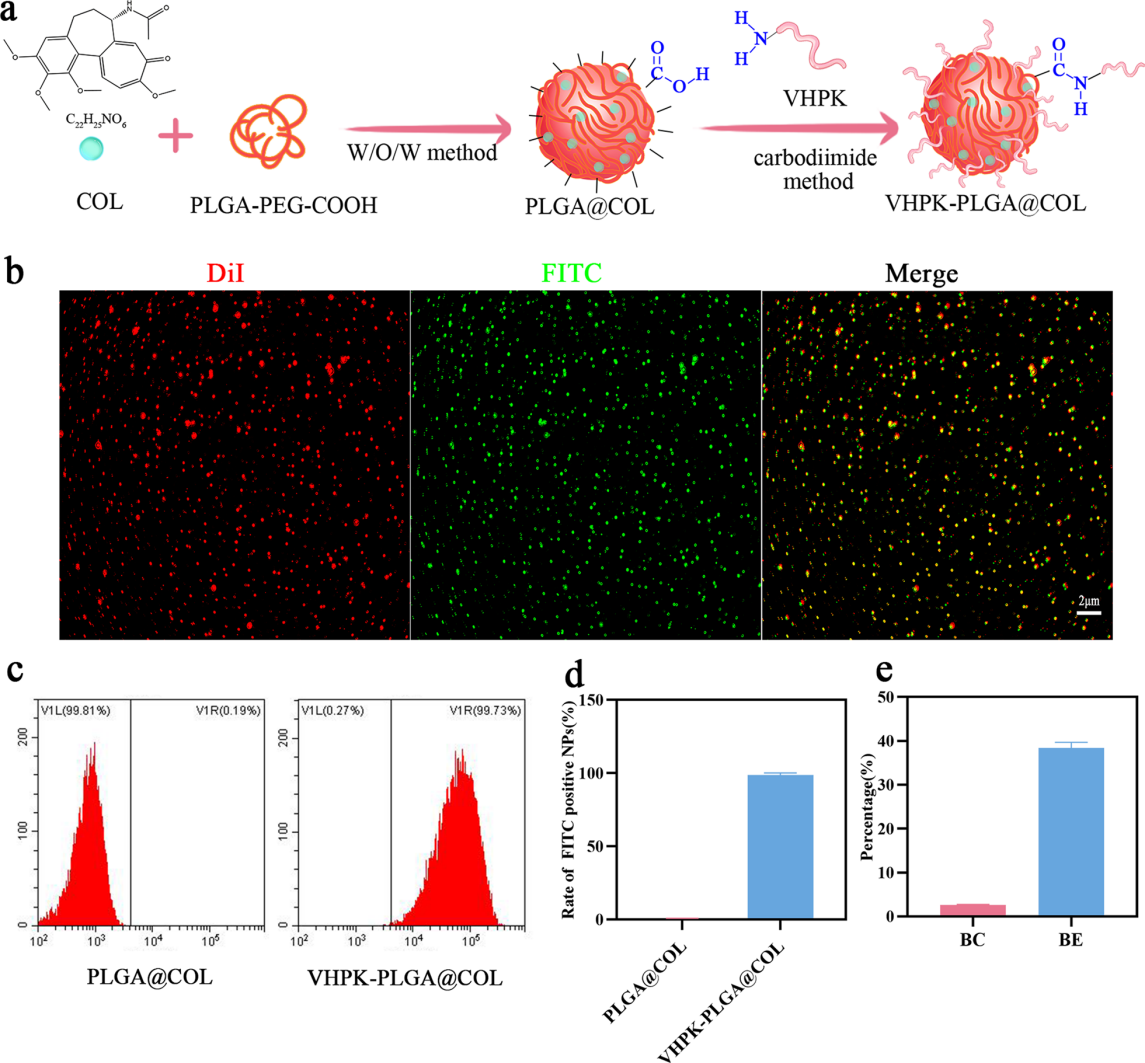
Preparation of VHPK-PLGA@COL and confirmation of VHPK peptide binding. **a** Schematic illustration of the preparation of VHPK-PLGA@COL. **b** Investigation of VHPK peptide (labeled with FITC in green) on the surface of PLGA@COL (labeled with DiI in red) by CLSM (scale bar = 2 μm). **c** Quantitative analyses of the conjugation rate of VHPK peptide on PLGA@COL and VHPK-PLGA@COL by the FCM method. **d** Quantitative analysis of flow cytometry. **e** The VHPK peptide binding capacity (BC) and binding efficiency (BE) on the surface of VHPK-PLGA@COL

PLGA@COL was synthesized using the improved W/O/W method. To achieve specific and precise targeting of inflammatory endothelial cells in atherosclerotic plaques, PLGA@COL was further modified with the VHPK peptide by forming an amide bond via a chemical reaction between the –COOH groups of PLGA@COL and the –NH_2_ groups of the VHPK peptide. After 12 h of incubation, dehydration condensation was performed. A schematic diagram of drug synthesis is illustrated in Fig. 2a. To confirm the successful conjugation of PLGA@COL with the VHPK peptide, images were obtained by CLSM, which showed the greatest overlap between green (FITC-VHPK) and red (PLGA@COL/DiI) in the nanoparticles (Fig. 2b). FCM quantitatively confirmed that the conjugation rate of the VHPK peptide was strikingly high at 98.72 ± 1.26% (Fig. 2c, d). Further studies showed that the VHPK peptide binding capacity and binding efficiency were 2.63 ± 0.07% and 38.38 ± 1.31%, respectively (Fig. 2e).

### Characterization of VHPK-PLGA@COL and drug release

#### Characterization of VHPK-PLGA@COL

**Fig. 3:**
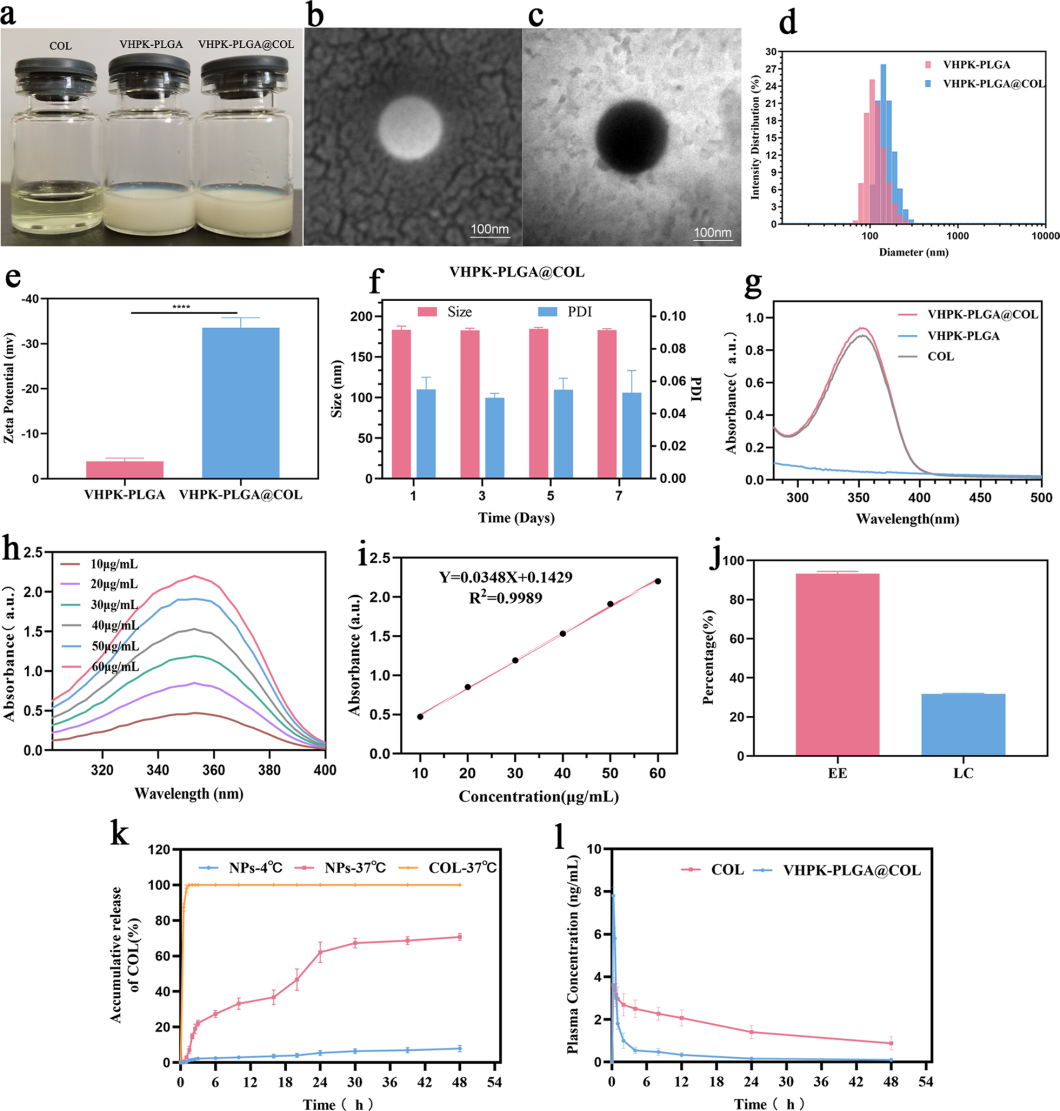
Characterization of VHPK-PLGA@COL and drug release. **a** Appearance characteristics of COL, VHPK-PLGA and VHPK-PLGA@COL. **b**, **c** SEM and TEM images of VHPK-PLGA@COL. **d**, **e** The size distribution and zeta potentials of VHPK-PLGA and VHPK-PLGA@COL. **f** Average size and PDI of VHPK-PLGA@COL over 7 days. **g** UV‒vis–NIR absorbance spectra of VHPK-PLGA, COL and VHPK-PLGA@COL. **h** The absorbance of COL at different concentrations. **i** The corresponding concentration-absorbance intensity relationship at a wavelength of 353 nm. (**j**) The encapsulation efficiency (EE) and drug loading capacity (LC) of VHPK-PLGA@COL. **k** In vitro cumulative release of COL from VHPK-PLGA@COL in PBS solution at 4 °C and 37 °C, 100 rpm, and COL in PBS solution at 37 °C, 100 rpm. **l** Plasma concentration–time profiles of colchicine in rats after intravenous administration of colchicine and VHPK-PLGA@COL at an equivalent dose of 0.1 mg/kg colchicine. (mean ± SD, n = 3)

We prepared VHPK-PLGA@COL, VHPK-PLGA, and colchicine solutions at the same concentration in PBS. The free colchicine solution appeared as a light yellowish-green clear liquid, while the VHPK-PLGA@COL and VHPK-PLGA solutions were milky white (Fig. 3a). The SEM and TEM images further confirmed the well-defined spherical morphology of VHPK-PLGA@COL (Fig. 3b-c). The diameters of VHPK-PLGA and VHPK-PLGA@COL were 155.97 ± 1.80 nm and 187.50 ± 1.71 nm, respectively (Fig. 3d), which were consistent with the TEM and SEM analyses. The zeta potentials of VHPK-PLGA and VHPK-PLGA@COL were − 3.89 ± 0.58 mV and − 33.56 ± 1.82 mV, respectively (Fig. 3e). Moreover, the average size and PDI of VHPK-PLGA@COL did not change significantly over 7 d, thus confirming the good stability of the nanoparticles (Fig. 3f).

The UV–vis–NIR spectrum showed that VHPK-PLGA@COL and colchicine featured a characteristic absorption peak at 353 nm, but no such peak was exhibited for VHPK-PLGA (Fig. 3g). These different features between VHPK-PLGA and VHPK-PLGA@COL implied that colchicine was successfully loaded in the nanoparticles. In addition, the absorbance of colchicine at different concentrations was measured by UV‒vis–NIR spectrophotometry (Fig. 3h), showing a linear relationship between the absorbance at 353 nm and colchicine concentration (Fig. 3i). According to the standard curve of colchicine constructed from the absorption spectra, the encapsulation efficiency and loading capacity of colchicine in VHPK-PLGA@COL were 93.32 ± 1.14% and 31.81 ± 0.27%, respectively (Fig. 3j).

### Sustained drug release by VHPK-PLGA@COL

To evaluate the cumulative drug release profile of colchicine, VHPK-PLGA@COL was transferred to dialysis bags and immersed in PBS (pH = 7.4) at 37 °C and 4 °C with shaking at 100 rpm, respectively, whereby an equivalent amount of free colchicine transferred to dialysis bags (37 °C, 100 rpm) served as the control. After 48 h, the amount of colchicine released in the nanoparticle groups at 37 °C and 4 °C were 70.73 ± 1.54% and 7.55 ± 0.79%, respectively, while the release rate in the free colchicine group reached 100% only after 1 h. As shown in Fig. 3k, the release of colchicine from VHPK-PLGA@COL was much slower at the storage temperature (4 °C) than that at body temperature (37 °C), whereas the release of colchicine from VHPK-PLGA@COL was also much slower than the free colchicine (70.73 ± 1.54% in 48 h *vs* 100% in 1 h), which might be attributed to the gradual degradation and rupture of PLGA and PEG polymers.

Following intravenous administration of free colchicine, the blood concentration of colchicine peaked rapidly and then decreased sharply with a maximum concentration of 7.82 ± 0.33 ng/mL, whereas the maximum concentration was 3.61 ± 0.55 ng/mL following intravenous administration of VHPK-PLGA@COL and a persistent and stable plasma drug concentration was observed over 48 h (Fig. 3l). These results demonstrated a steady and long-term colchicine release pattern from VHPK-PLGA@COL in vivo.

### In vitro cytotoxicity and blood compatibility of VHPK-PLGA@COL

**Fig. 4:**
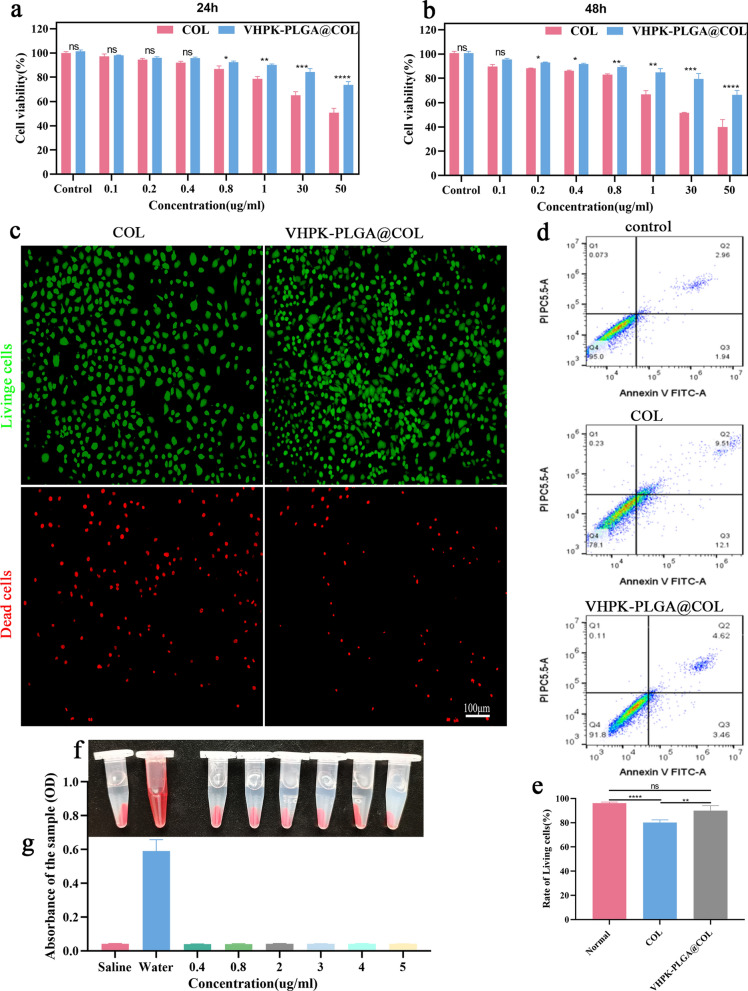
In vitro cytotoxicity and blood compatibility analyses of VHPK-PLGA@COL. **a**, **b** CCK-8 assay on cell viability of HUVECs after treatment with COL and VHPK-PLGA@COL at various concentrations for 24 h and 48 h, respectively. **c** Live/dead cell-staining images of HUVECs after treatment for 24 h with COL and VHPK-PLGA@COL (colchicine: 0.8 µg/mL) as observed by CLSM. **d**, **e** FCM analysis of the rate of living HUVECs after treatment with COL and VHPK-PLGA@COL (colchicine: 0.8 µg/mL) for 24 h. **f** Images of the hemolysis test of VHPK-PLGA@COL at different concentrations after centrifugation. **g** Measurement of the absorbance of the supernatant at 540 nm in the hemolysis test. (n = 3, *P < 0.05, **P < 0.01***P < 0.001, ****P < 0.0001; ns = no significance)

CCK-8, FCM and live/dead cell staining were employed to evaluate the cytotoxicity of VHPK-PLGA@COL. According to the CCK-8 cytotoxicity assay, colchicine and VHPK-PLGA@COL induced a dose-dependent response of cytotoxicity, whereby colchicine reduced cell viability at 24 h from 97.2% to 50.6% and VHPK-PLGA@COL reduced cell viability from 97.9% to 73.6% at doses ranging from 0.1 to 50 μg/mL (Fig. 4a). Following treatment for 48 h, cell viability decreased from 89.6% to 39.9% by colchicine and from 95.5% to 66.3% by VHPK-PLGA@COL, respectively (Fig. 4b). Based on these results, colchicine at a concentration of 0.8 µg/mL was used for the following live/dead cell staining and FCM assays. As shown in Fig. 4c, the live/dead cell-staining showed that the prevalence of green-fluorescent cells (live) in the VHPK-PLGA@COL group was obviously higher than that in the colchicine group. In addition, FCM analysis showed that the percentages of living cells in the normal control group, colchicine, and VHPK-PLGA@COL treatment group were 95.0%, 78.1%, and 91.8%, respectively (Fig. 4d, e), suggesting that colchicine loaded in VHPK-PLGA@COL was much less toxic to cells than free colchicine (cell viability 91.8% *vs* 78.1%) due to its encapsulation and sustained release from VHPK-PLGA@COL.

Hemocompatibility is an important evaluation criterion for the biosafety of biological materials. As shown in Fig. 4f, g, similar to saline, VHPK-PLGA@COL at various concentrations did not cause any hemolytic reactions, whereas red blood cells were obviously destroyed in the water. These results confirmed the good biocompatibility of VHPK-PLGA@COL, thus allowing further functional evaluation of these nanoparticles in vivo.

### Targeting ability of VHPK-PLGA@COL

#### Targeting ability of VHPK-PLGA@COL in vitro

**Fig. 5:**
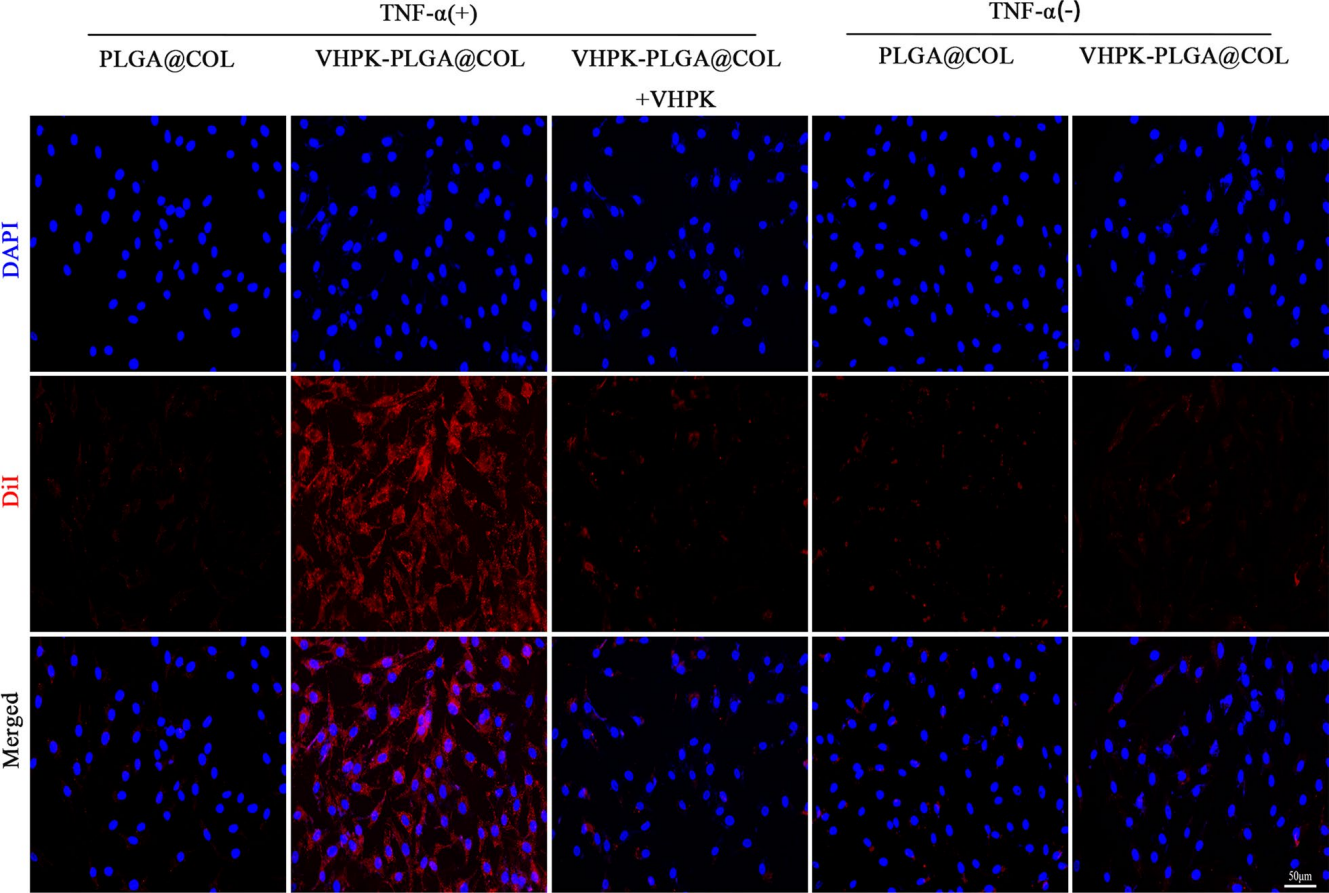
Targeting ability of PLGA@COL and VHPK-PLGA@COL in vitro. HUVECs with or without activation by TNF-α, TNF-α ( +) or TNF-α (-), were incubated with DiI-labeled PLGA@COL or VHPK-PLGA@COL for 2 h and images were observed with CLSM. For blocking analysis, activated cells were first treated with VHPK peptide solution followed by incubation with VHPK-PLGA@COL (middle column). (blue = nucleus, red = nanoparticles, scale bar = 50 μm)

The ability of VHPK-PLGA@COL labeled with DiI to target inactivated and TNF-α-activated HUVECs was observed using CLSM (Fig. 5). A substantial amount of VHPK-PLGA@COL was found to adhere to activated cells as presented by strong red fluorescence signals compared with the weak fluorescence signal in the PLGA@COL group. Blocking analysis showed that the addition of the VHPK peptide prior to the administration of VHPK-PLGA@COL to cells significantly weakened the fluorescence signals on targeted cells, thus verifying the specific function of VHPK peptide for endothelial cell targeting. Nevertheless, weak fluorescence was observed in inactivated cells following the administration of either VHPK-PLGA@COL or PLGA@COL. These results demonstrated that VHPK-PLGA@COL, not PLGA@COL, could specifically target the inflammation-activated HUVECs via the VHPK peptide.

**Fig. 6:**
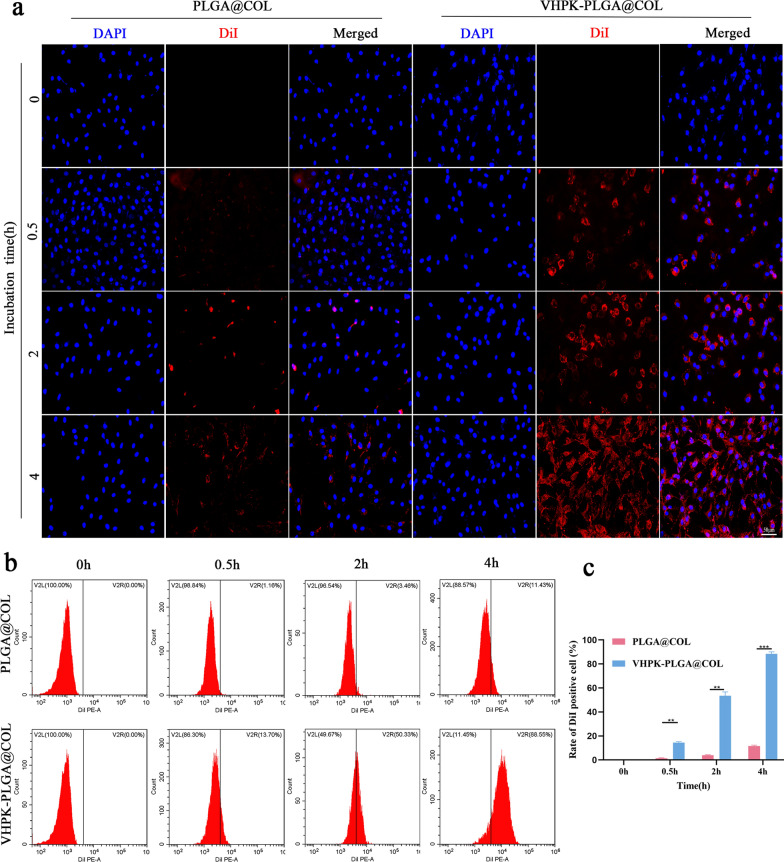
Targeting ability of VHPK-PLGA@COL in vitro: Time-point study. **a** CLSM images and **b** FCM results of activated HUVECs following incubation with DiI-labeled PLGA@COL or VHPK-PLGA@COL for different periods. **c** Quantitative results of data from flow cytometric analyses. (blue = nucleus, red = nanoparticles, scale bar = 50 μm, n = 3, **P < 0.01***P < 0.001)

To further determine the binding efficiency, activated HUVECs were incubated with VHPK-PLGA@COL or PLGA@COL for different periods. The results showed that a few nanoparticles accumulated around the cells at 0.5 h and some nanoparticles appeared around the nucleus at 2 h after incubation with VHPK-PLGA@COL. At 4 h, many nanoparticles were swallowed by the cells and gathered around the nucleus. Remarkably, the fluorescence intensity gradually increased in cells incubated with VHPK-PLGA@COL, while it became less obvious in cells incubated with PLGA@COL (Fig. 6a). These findings were further confirmed by FCM analyses (Fig. 6b, c).

#### In vivo targeting of atherosclerotic plaques by VHPK-PLGA@COL

**Fig. 7:**
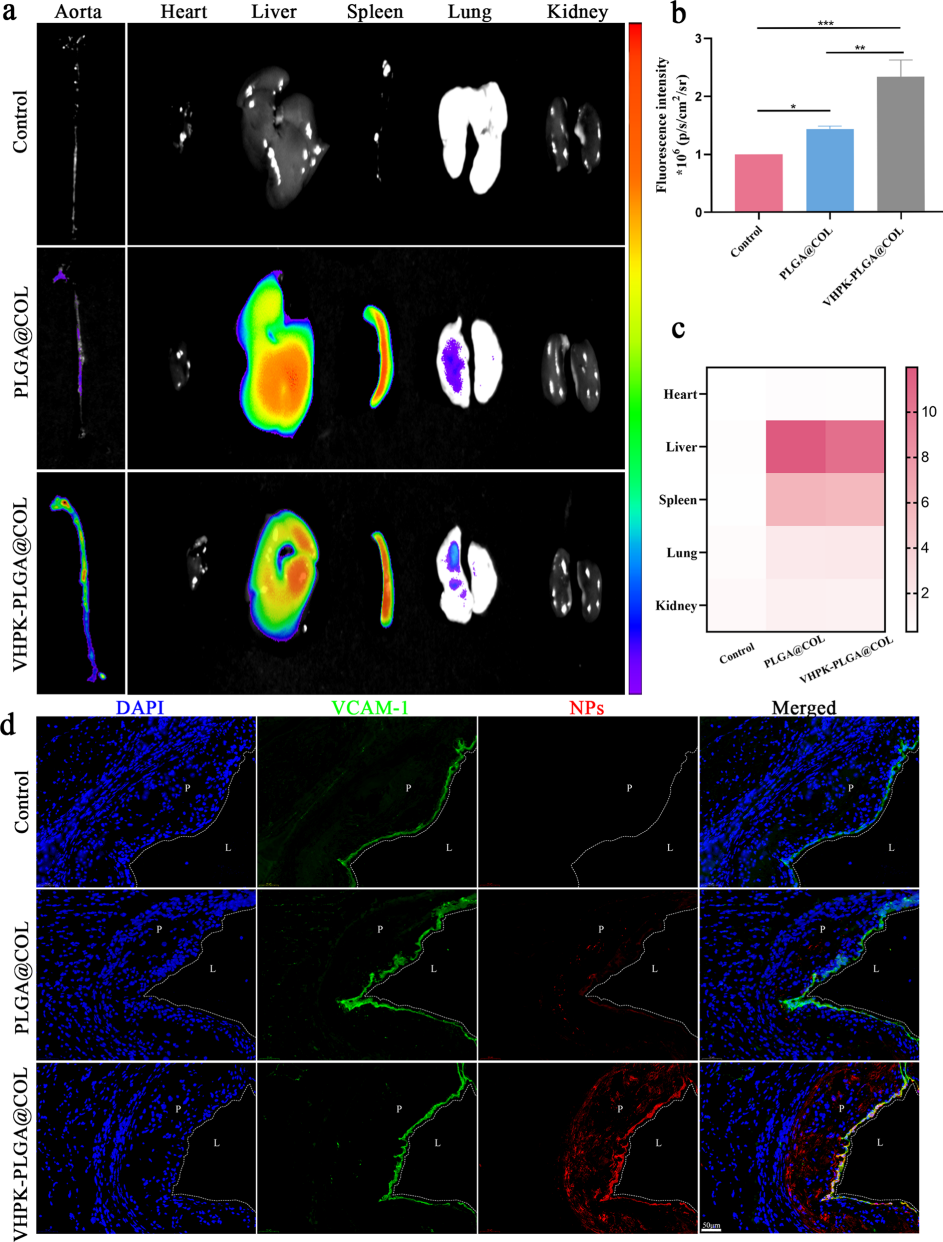
Targeting atherosclerotic plaques by VHPK-PLGA@COL in vivo. **a** Representative ex vivo fluorescence images of aortas and major organs, **b** Quantitative data of DiR fluorescent signals accumulated in the aortas, **c** Heatmap of DiR fluorescent signals in major organs at 24 h post intravenous injection (n = 3, *P < 0.05, **P < 0.01, ***P < 0.001). **d** CLSM images of aortic sinus sections prepared from atherosclerotic mice after intravenous injection of saline, DiR-labeled VHPK-PLGA or VHPK-PLGA@COL for 24 h. (blue = DAPI, green = VCAM-1, red = nanoparticles, P = atherosclerotic plaque, L = vessel lumen, scale bar = 50 μm)

To investigate the ability of VHPK-PLGA@COL to target atherosclerotic lesions and its pharmacokinetics, VHPK-PLGA@COL and PLGA@COL labeled with DiR were intravenously injected into mice with HCD-induced atherosclerosis, while mice injected with saline served as the control. Since the surface of the atherosclerotic plaques was featured with inflamed endothelial cells overexpressing VCAM-1, it is then supposed to be the specific target of VHPK-PLGA@COL. As expected, ex vivo imaging of the harvested aortas from mice injected with VHPK-PLGA@COL harbored much stronger fluorescence signals than those from PLGA@COL group (Fig. 7a, b). Consistently, imaging analysis of aortic sinus sections further confirmed that the VHPK-PLGA@COL-treated plaques had significantly higher DiR fluorescence signals than the PLGA@COL-treated plaques (Fig. 7d). These data demonstrated that VHPK-PLGA@COL was capable of binding to atherosclerotic plaques at a considerably higher level than PLGA@COL due to the unique property of VHPK peptide to target VCAM-1 molecules overexpressed on inflammatory endothelial cells.

In addition, the distribution of DiR fluorescence signals in certain major organs, especially the liver and spleen, was also evaluated. Compared to PLGA@COL without the inflammatory endothelial cell targeting capacity, the unexpected distribution of VHPK-PLGA@COL in the major organs slightly decreased (Fig. 7a, c), thus providing additional possibilities to target atherosclerotic plaques for VHPK-PLGA@COL.

### Evaluation of the therapeutic efficacy of VHPK-PLGA@COL in vivo

#### In vivo biosafety evaluation

**Fig. 8:**
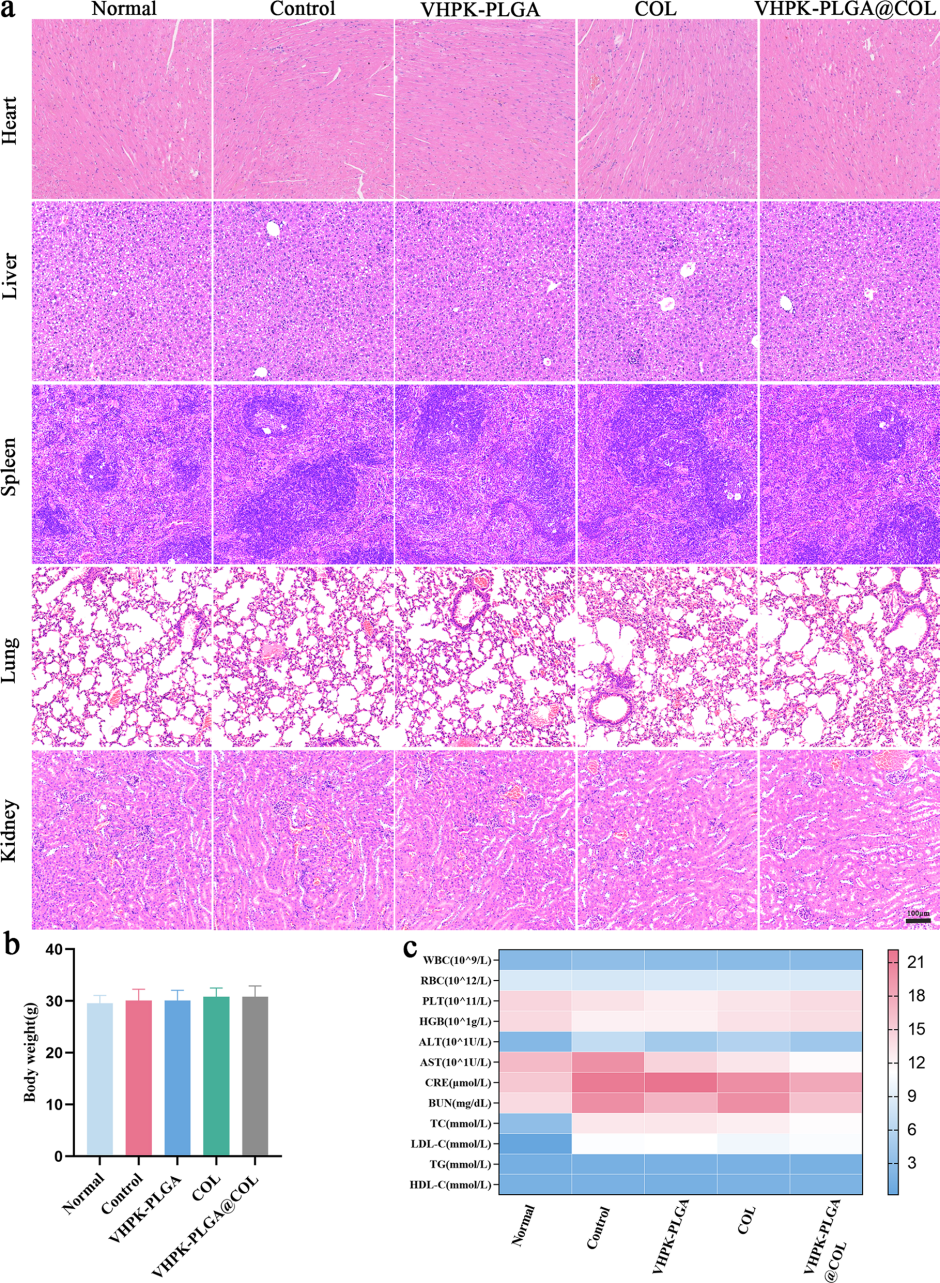
Biosafety evaluation of VHPK-PLGA@COL in vivo. **a** H&E staining of the heart, liver, spleen, lung, and kidney of ApoE − / − mice after different treatments. **b** Body weight and **c** hematological parameters and biochemical parameters of ApoE − / − mice after different treatments. (n = 5, Scale bar = 100 µm)

Next, various approaches, including body weight examination, hematological analysis, blood biochemical analysis, and histological analysis of main organs by H&E staining, were employed to systemically evaluate the biosafety of VHPK-PLGA@COL. Histologically, H&E staining showed no obvious histological changes in the main organs of mice from five different groups (Fig. 8a). Meanwhile, no significant difference was observed in the body weight of mice from all 5 groups (Fig. 8b). Hematological analysis showed that there were no significant variations in RBCs, PLTs, or HGB. Importantly, the counts of WBCs, the immune-associated cells, were similar between the different treatment groups and the untreated normal group of mice (Fig. 8c). Additionally, clinical biochemical analysis showed normal levels of ALT, AST, CRE, and BUN, indicating that the biological functions of the liver and kidney were not affected by systemic administration of nanoparticles (Fig. 8c). On the other hand, the levels of TC and LDL-C in mice from the non-treated control, VHPK-PLGA, COL, and VHPK-PLGA@COL groups all consistently increased due to HCD with no significant differences among these different groups of atherosclerotic mice. Nevertheless, TG and HDL-C remained at normal levels and showed no significant differences among all groups of mice (Fig. 8c). Taken together, these results have demonstrated the good biocompatibility and safety of these nanoparticles in vivo and colchicine had no significant effect on plasma lipid metabolism [33, 34].

#### In vivo anti-atherosclerotic effect of VHPK-PLGA@COL

##### Quantitative analysis of atherosclerotic plaques

**Fig. 9:**
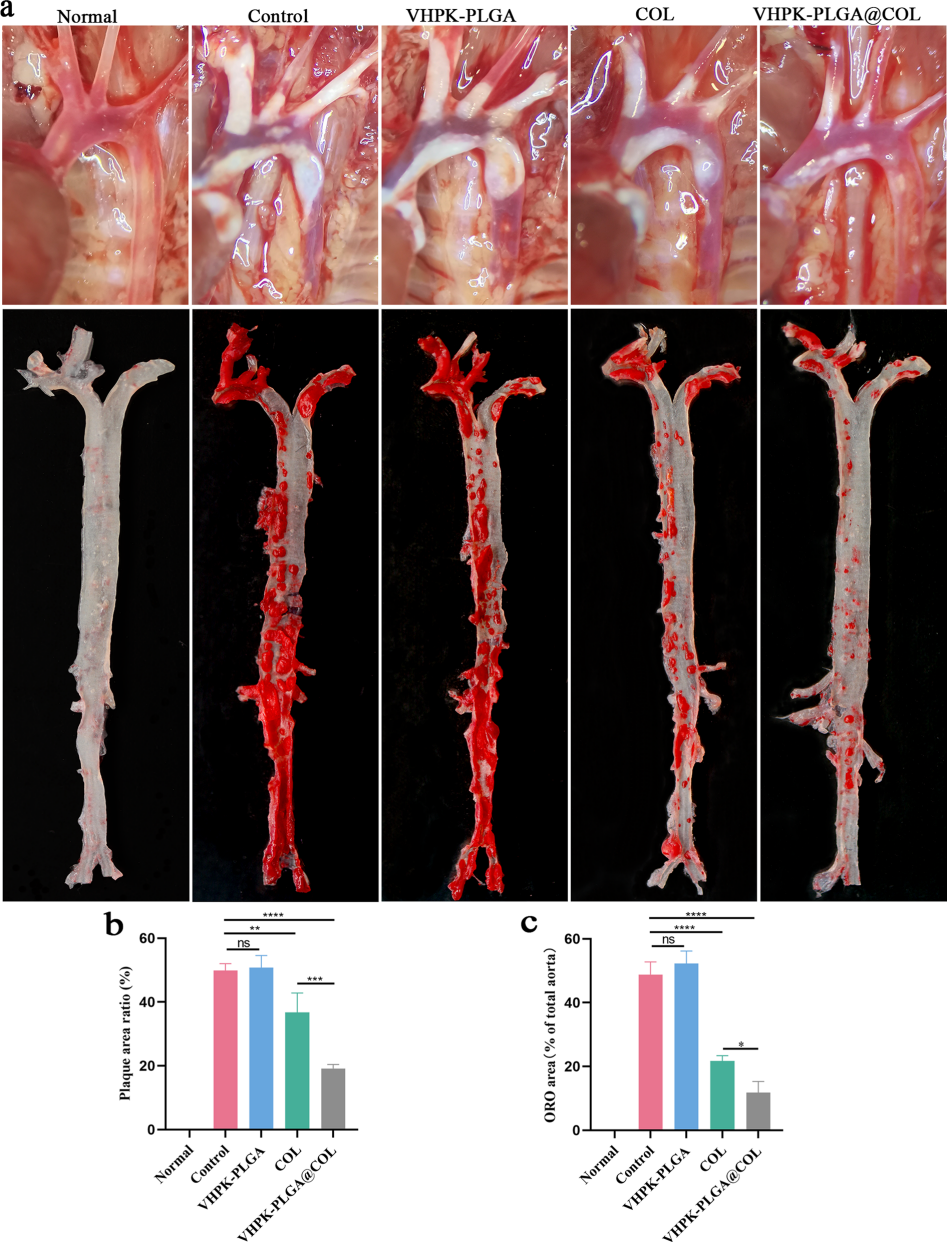
Quantitative analysis of atherosclerotic plaques. **a** Digital images of the aortic arch (top) and en face micrographs of ORO-stained aortas (bottom) after 8 weeks of treatment. **b** Plaque area ratio (%) in the aortic arch; (**c**) Quantitation of the percentage of ORO-positive area compared with total luminal surface area (n = 5, *P < 0.05, **P < 0.01***P < 0.001, ****P < 0.0001; *ns*  no significance)

We next examined the therapeutic efficacy of different formulations against AS development. As shown in the digital images of the aortic arch (Fig. 9a), VHPK-PLGA@COL significantly decreased the plaque area (19.16 ± 1.02%), whereas free colchicine only slightly reduced the plaque area (36.76 ± 4.93%) compared with the non-treated control group (49.86 ± 1.78%) (Fig. 9b). Similarly, the en face micrographs of the ORO-stained aortas showed that treatment with VHPK-PLGA@COL and colchicine reduced the plaque areas of the entire aortas from 48.82 ± 3.24% to 11.82 ± 2.81% and 21.75 ± 1.34% (Fig. 9c), respectively. Even though both colchicine and VHPK-PLGA@COL exhibited anti-atherosclerotic effects, the therapeutic efficacy of VHPK-PLGA@COL was more pronounced than that of free colchicine, possibly due to their enhanced enrichment in the targeted site.

##### Assessing the effect of VHPK-PLGA@COL on the stability of the atherosclerotic plaques

The unpredictable rupture of vulnerable atherosclerotic plaques triggers adverse cardiovascular events such as acute coronary syndrome (ACS) and even sudden cardiac death (SCD) [35]. Therefore, assessing the stability of atherosclerotic plaques is of significance in plaque risk stratification and the reduction of major adverse cardiovascular events (MACEs). Herein, we further assessed the effect of VHPK-PLGA@COL on the stability of atherosclerotic plaques by examining the changes in the pathological features and biological components within the plaque following different treatments.

**Fig. 10:**
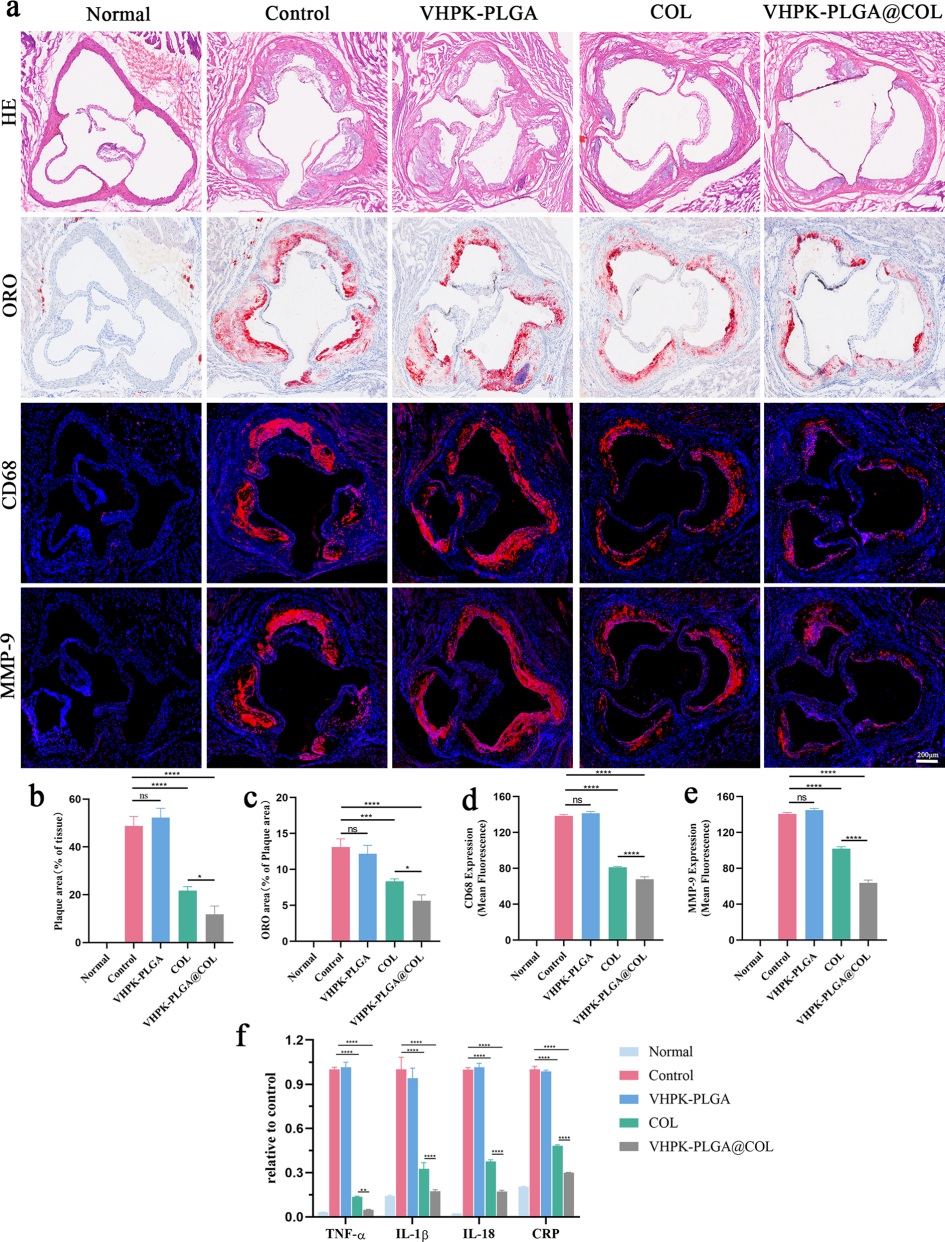
Assessment of the effect of VHPK-PLGA@COL on the stability of atherosclerotic plaques and measurement of plasma inflammatory cytokines in mice. **a** H&E and ORO-staining, and immunofluorescence studies on the expression of CD68 and MMP-9 on cross-sections of aortic sinus (scale bar = 200 μm). **b-e** Quantitative analysis of plaque area, lipid deposition area, and CD68 and MMP-9 mean fluorescence intensity in cross-sections of the aortic sinus. **f** The levels of proinflammatory factors (TNF-α, IL-1β, IL-18 and CRP) in the plasma of mice from different treatment groups were measured using ELISAs. (n = 5, *P < 0.05, **P < 0.01, ***P < 0.001, ****P < 0.0001; *ns*  no significance)

Histologically, H&E staining of the cross-sections of the aortic sinus showed that the plaque area dramatically decreased after treatment with colchicine (21.75 ± 1.34%) or VHPK-PLGA@COL (11.82 ± 2.81%) compared with the non-treated control group (48.82 ± 3.24) (Fig. 10a,b). Similarly, the ORO area was significantly decreased in the colchicine (8.36 ± 0.26%) and VHPK-PLGA@COL (5.65 ± 0.66%) groups compared with the non-treated control group (13.10 ± 0.94%) (Fig. 10a, c). Consistently, these results indicated that VHPK-PLGA@COL exhibited a better therapeutic effect on AS than free colchicine according to plaque burden and lipid deposition analyses.

Macrophages are the main immune cell components within atherosclerotic plaques and play critical roles in the occurrence and development of AS. Macrophages progressively engulf ox-LDL and serve as major sources of foam cells. Additionally, macrophages secrete cytokines and chemokines that interact with other immune cells and contribute to inflammatory cascade reactions and plaque rupture [36, 37]. In addition, MMP-9 plays an important role in extracellular matrix degradation, a process that leads to atherosclerotic plaque destabilization [33]. Hence, we examined the effect of VHPK-PLGA@COL on macrophage infiltration and MMP9 expression in atherosclerotic plaques. Immunofluorescence (IF) staining of CD68 (a macrophage marker) on aortic sinus sections showed that treatment with colchicine and VHPK-PLGA@COL led to a 41.32% and 50.97% reduction in the number of macrophages, respectively, compared with the non-treated control group (Fig. 10a, d). On the other hand, IF immunostaining of MMP9 showed that treatment with colchicine and VHPK-PLGA@COL decreased the expression of MMP-9 by 27.60% and 54.68%, respectively, compared with the non-treated control group (Fig. 10a, e). In general, the AS plaques in the VHPK-PLGA@COL group exhibited a stable phenotype as featured by the smallest plaque burden, the least lipid deposition, the fewest infiltration of macrophages, and the lowest expression of MMP-9 compared with colchicine-treated and non-treated control groups.

#### Quantification of the inflammatory cytokines in plasma

We then examined the levels of several inflammatory cytokines (TNF-α, IL-1β, IL-18 and CRP) in the plasma from mice following different treatments for 8 weeks. As shown in Fig. 10f, systemic administration of colchicine and VHPK-PLGA@COL significantly reduced the plasma levels of these inflammatory cytokines compared to non-treated group, suggesting that the anti-atherosclerotic effect of colchicine and VHPK-PLGA@COL might be associated with their potent anti-inflammatory functions.

### The potential mechanisms involved in VHPK-PLGA@COL-mediated anti-atherosclerotic effect

**Fig. 11:**
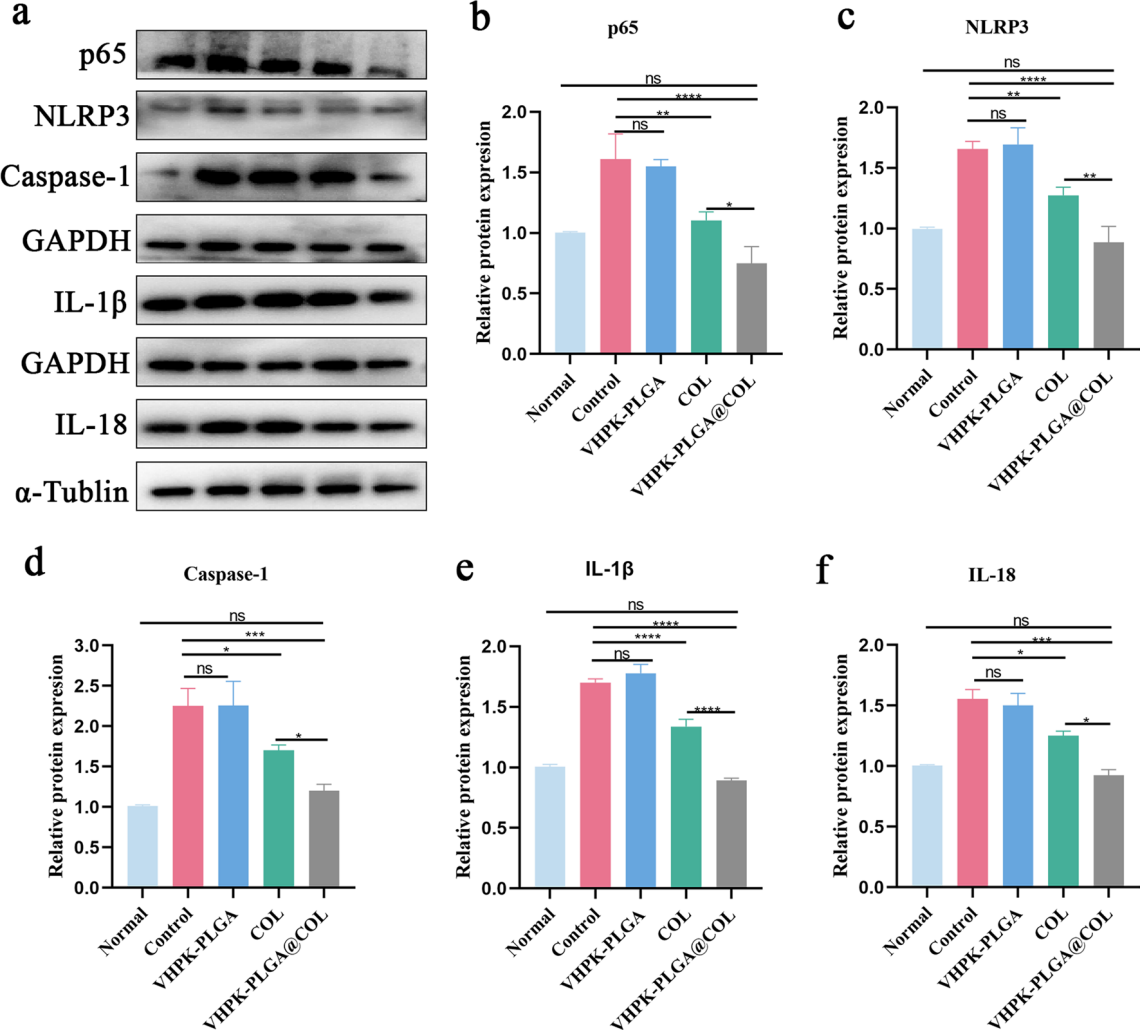
Inhibitory effect of VHPK-PLGA@COL on NF-κB/NLRP3 inflammasome pathways. **a** Western blot (WB) analysis on the expression of p65, NLRP3, caspase-1, IL-1β, and IL-18 proteins in the aortas of different groups of mice after 8 weeks of treatment. **b**-**f** The semi-quantification of WB data on p65, NLRP3, caspase-1, IL-1β, and IL-18 protein expressions in aorta (n = 5, *P < 0.05, **P < 0.01, ***P < 0.001, ****P < 0.0001, *ns*  no significance)

The NLRP3 inflammasome constitutes one of the important immune response cascades and plays a crucial role in the formation and progression of AS [28, 38, 39]. The NLRP3 inflammasome complex is composed of NLRP3, apoptosis-associated speck-like protein (ASC), and procaspase-1, whereby NF-κB plays an essential role in the first step of NLRP3 inflammasome activation through regulating the expression of the NLRP3 protein and some inflammatory cytokine precursors (e.g., pro-IL-1β and pro-IL-18). Herein, we explored whether colchicine and VHPK-PLGA@COL achieved their therapeutic effects on AS through interfering with the NF-κB/NLRP3 pathways. As expected, the levels of NF-κB p65, NLRP3, caspase-1, IL-1β and IL-18 remained relatively low in aortas of healthy normal mice, but markably increased in the aortas of both non-treated and VHPK-PLGA-treated AS mice, suggesting that the elevated activation of the NF-κB/NLRP3 inflammasome pathway is closely associated with AS development and that VHPK-PLGA had no inhibitory effect on NF-κB/NLRP3 inflammasome activation. On the contrary, treatment with colchicine and VHPK-PLGA@COL significantly decreased the levels of these NF-κB/NLRP3 inflammasome components in the aortic lesions of AS mice. Again, the inhibitory effect of VHPK-PLGA@COL on the NF-κB/NLRP3 inflammasome pathway was more pronounced than that of free colchicine(Fig. 11a–f). Taken together, these results suggest that colchicine's anti-atherosclerotic effect was possibly attributed to the inhibition of NF-κB/NLRP3 inflammasome activation, which was further amplified by VHPK-PLGA@COL due to their unique properties of inflammatory endothelial cell targeting and sustained release.

## Discussion

In recent years, emerging evidence from several clinical trials (LoDoCo, LoDoCo2, COLCOT and COPS trials) strongly suggests that clinical application of colchicine is associated with significant reductions in the incidence of the composite of cardiovascular mortality [40]. However, it has not yet been approved for anti-atherosclerosis in clinic. The primary barrier for the clinical translation of colchicine in anti-atherosclerosis treatment is its adverse effects after systemic administration. Pharmacologically, colchicine is metabolized by the CYP3A4 enzyme and cleared by the P-glycoprotein efflux pump in the bile and kidneys. It has been shown that potent inhibitors of the CYP3A4 and P-glycoprotein efflux pathways or the malfunction of the liver and kidney can increase blood drug concentration and even cause acute colchicine toxicity [40]. However, polypharmacy and the related comorbidities, especially renal dysfunction, are common phenomena among AS patients [41] that can increase the possibility of colchicine-related adverse effects. These limitations prompted us to reconsider a new strategy to repurpose colchicine for anti-atherosclerosis treatment and concomitantly minimize its systemic toxicity. To date, there is still lack of related studies on how to reduce colchicine toxicity. In the present study, we aimed to develop a colchicine-loaded nanoparticle platform that can preferably deliver the drug to atherosclerotic plaque sites with sustained release and reduced systemic toxicity.

PLGA, one of the most successfully developed biodegradable polymers [42], has been approved by FDA and widely utilized owing to its properties of excellent biocompatibility, biodegradability, and controlled drug release[25]. The safety of PLGA has been well established in previous studies[43–45], and this polymer is currently used in resorbable sutures, bone implants, and tissue engineering scaffolds in humans[46]. Meanwhile, PLGA presents a stable linker to polyethylene glycol to improve the circulation and half-life in the blood and to couple targeting moieties for specific cellular uptake [25, 47]. These unique properties of PLGA make them good candidates for fabricating nanoparticles as efficient carriers of drugs, including colchicine. Herein, we successfully fabricated PLGA-based nanoparticles encapsulated with colchicine, designated as VHPK-PLGA@COL, and confirmed that these nanoparticles achieved sustained drug release (Fig. 3k–l) and attenuated the cell toxicity of colchicine (Fig. 4a–g) both in vitro and in vivo. Meanwhile, after systemic administration of VHPK-PLGA@COL into AS mice for 8 weeks, no obvious abnormal changes were observed in body weight, hematological and blood biochemical parameters, and the histology and function of several main organs, thus further confirming their biosafety and excellent biocompatibility in mice (Fig. 8a–c).

Endothelial cells lining the inner layer of the blood vessels are in direct contact with the blood, thus enabling nanoparticles to easily target inflamed endothelium on atherosclerotic plaques. Endothelial cells are vulnerable to becoming inflamed and dysfunctional because of their constant exposure to various risk factors, such as hypercholesterolemia, hyperglycemia, hypertension, and smoking, and then play a critical role in the initiation and progression of AS. Hence, targeted inhibition of inflammatory endothelial cells is conducive to the early control or therapy of AS. In the present study, we made functionalized VHPK-PLGA@COL modified with the VHPK peptide which can specifically target VCAM-1, a receptor that is usually overexpressed by inflamed endothelial cells on atherosclerotic plaques [48], making it an attractive candidate target molecule for endothelial-specific drug delivery in the treatment of AS. In previous studies, various types of nanoparticles have been conjugated with VHPK peptide and exhibited good targeting capability and anti-atherosclerotic effects [48–50]. Herein, we have demonstrated that VHPK-PLGA@COL can preferably accumulate in inflamed endothelial cells both in vitro and in vivo as compared to the nontargeted NPs (PLGA@COL) (Figs. 5–7). In the AS mouse model, we showed that systemic administration of free colchicine and VHPK-PLGA@COL significantly reduced plaque areas and enhanced plaque stability compared to the non-treated AS mice, but VHPK-PLGA@COL was significantly enriched in atherosclerotic plaques and exhibited augmented anti-atherosclerotic efficacy compared with free colchicine (Figs. 9–10). These compelling findings suggest that colchicine encapsulated in VHPK-PLGA@COL could be optimally tuned to maximize their therapeutic efficacy while minimizing their adverse effects in anti-atherosclerotic treatment due to their unique capability to target inflamed endothelial cells.

The repurposing of colchicine for the treatment of atherosclerotic cardiovascular diseases will benefit from a better understanding of how it works. The NF-κB/NLRP3 activation involves two key steps, priming and activation (Fig. 1b). During priming, various stimuli activate NF-κB signaling through damage-associated molecular patterns (DAMPs), leading to increased expression of pro-IL-1β, pro-IL-18, NLRP3, ASC and procaspase-1. In the second step, further stimuli promote the assembly of the NLRP3 inflammasome complex, which subsequently induces the splicing of procaspase-1 itself to obtain activated caspase-1 responsible for cleaving pro-IL-1β and pro-IL-18 into the mature and secreted forms IL-1β and IL-18 [51]. Extracellular IL-1β and IL-18 trigger a cascade of inflammatory responses and amplify inflammation through self-activation [52]. These changes facilitate the recruitment of monocytes/macrophages and increase vascular inflammation, which in turn further activate the NF-κB/NLRP3 pathways. This vicious circle most likely contributes to the non-resolving chronic vascular inflammation that drives AS progression. Indeed, several lines of evidence have shown that NF-κB/NLRP3 inflammasome pathways play an important role in AS development and progression [9, 53, 54]. A previous study has shown that colchicine exhibited therapeutic effect on AS through inhibiting the assembly of the NLRP3 inflammasome by binding tubulin to disturb microtubule dynamics, leading to the inhibition of the cleavage of pro-IL-1β and pro-IL-18 into mature forms and the subsequent attenuation of inflammation in the vessel wall [55]. In the present study, we demonstrated a significantly elevated level of several components (NF-κB p65, NLRP3, caspase-1, IL-1β and IL-18) in NF-κB/NLRP3 pathways in atherosclerotic plaques, while systemic administration of free colchicine and VHPK-PLGA@COL into AS mice significantly downregulated the expression level of these inflammatory components, whereby the downregulation of NF-κB may play a central role in colchicine-mediated inhibition of NLRP3 inflammasome pathways. Consistently, our results indicate that VHPK-PLGA@COL-mediated inhibition of NF-κB/NLRP3 pathways was more pronounced than free colchicine treatment. Taken together, these findings suggest that VHPK-PLGA@COL exerted potent anti-atherosclerotic effects possibly through their augmented inhibition of NF-κB/NLRP3 pathways.

## Conclusion

In this study, PLGA@COL was synthesized by an improved W/O/W method, and the targeting moieties (VHPK peptide) were attached to the surface of PLGA@COL to form VHPK-PLGA@COL. VHPK-PLGA@COL has significantly improved the biosafety and therapeutic effects of colchicine in AS mouse models because of its encapsulation and sequentially sustained release from nanoparticles with unique capability to target inflamed endothelial cells. Mechanistically, the improved anti-atherosclerotic effects conferred by VHPK-PLGA@COL are most likely attributed to their augmented inhibitory effects on the NF-κB/NLRP3 pathways. These compelling findings have shown great potential for the translational repurposing of colchicine delivered through VHPK-PLGA@COL in anti-atherosclerotic treatment.

## Data Availability

All data generated or analysed during this study are included in this published article.

## Abbreviations

CVD: Cardiovascular disease
AS: Atherosclerosis
VCAM-1: Vascular cell adhesion molecule-1
CAD: Coronary artery disease
COL: Colchicine
NLRP3: Nod-like receptor family pyrin domain containing protein 3
NF-κB: Nuclear factor–κB
PLGA: Poly(lactic-co-glycolic acid)
PEG: Poly(ethylene glycol)
NPs: Nanoparticles
ApoE − / −: Apolipoprotein-E-deficient mice
PVA: Polyvinyl alcohol
DAPI: 4′,6-Diamidino-2-phenylindole
ORO: Oil red O
H&E: Hematoxylin and eosin
HUVECs: Human umbilical vein endothelial cells
CLSM: Confocal laser scanning microscopy
FCM: Flow cytometry
SEM: Scanning electron microscopy
TEM: Transmission electron microscopy
PDIs: Polydispersity indexes
DLS: Dynamic light scattering
LC: Loading capacity
EE: Encapsulation efficiency
HCD: High-cholesterol diet
NCD: Normal chow diet
EDTA: Ethylenediaminetetraacetic acid
IVIS: In vivo imaging system
RBCs: Red blood cells
PLTs: Platelets
HGB: Hemoglobin
WBCs: White blood cells
ALT: Alanine aminotransferase
AST: Aspartate aminotransferase
CRE: Creatinine
BUN: Blood urea nitrogen
TC: Total cholesterol
TG: Triglyceride
LDL-C: Low-density lipoprotein cholesterol
HDL-C: High-density lipoprotein cholesterol
MMP-9: Matrix metalloproteinase-9
ACS: Acute coronary syndrome
SCD: Sudden cardiac death
MACEs: Major adverse cardiovascular events
ASC: Apoptosis-associated speck-like protein
DAMPs: Damage-associated molecular patterns

## References

[1] Virani S, Alonso A, Benjamin E, Bittencourt M, Callaway C, Carson A, Chamberlain A, Chang A, Cheng S, Delling F (2020). Heart disease and stroke statistics-2020 update: a report from the american heart association. Circulation.

[2] Ridker P, Bhatt D, Pradhan A, Glynn R, MacFadyen J, Nissen S (2023). Inflammation and cholesterol as predictors of cardiovascular events among patients receiving statin therapy: a collaborative analysis of three randomised trials. Lancet.

[3] Gimbrone M, García-Cardeña G (2016). Endothelial cell dysfunction and the pathobiology of atherosclerosis. Circ Res.

[4] Distasio N, Dierick F, Ebrahimian T, Tabrizian M, Lehoux S (2022). Design and development of Branched Poly(ß-aminoester) nanoparticles for Interleukin-10 gene delivery in a mouse model of atherosclerosis. Acta Biomater.

[5] Nahrendorf M, Jaffer FA, Kelly KA, Sosnovik DE, Aikawa E, Libby P, Weissleder R (2006). Noninvasive vascular cell adhesion molecule-1 imaging identifies inflammatory activation of cells in atherosclerosis. Circulation.

[6] Libby P (2002). Inflammation in atherosclerosis. Nature.

[7] Engelen S, Robinson A, Zurke Y, Monaco C (2022). Therapeutic strategies targeting inflammation and immunity in atherosclerosis: how to proceed?. Nat Rev Cardiol.

[8] Seijkens T, van Tiel C, Kusters P, Atzler D, Soehnlein O, Zarzycka B, Aarts S, Lameijer M, Gijbels M, Beckers L (2018). Targeting CD40-induced TRAF6 signaling in macrophages reduces atherosclerosis. J Am Coll Cardiol.

[9] Ridker PM, Everett BM, Thuren T, MacFadyen JG, Chang WH, Ballantyne C, Fonseca F, Nicolau J, Koenig W, Anker SD (2017). Antiinflammatory therapy with canakinumab for atherosclerotic disease. N Engl J Med.

[10] Biasucci L, Pedicino D, Liuzzo G (2020). Promises and challenges of targeting inflammation to treat cardiovascular disease: the post-CANTOS era. Eur Heart J.

[11] Sehested T, Bjerre J, Ku S, Chang A, Jahansouz A, Owens D, Hlatky M, Goldhaber-Fiebert J (2019). Cost-effectiveness of canakinumab for prevention of recurrent cardiovascular events. JAMA cardiology.

[12] Adler Y, Charron P, Imazio M, Badano L, Barón-Esquivias G, Bogaert J, Brucato A, Gueret P, Klingel K, Lionis C (2015). 2015 ESC Guidelines for the diagnosis and management of pericardial diseases: the task force for the diagnosis and management of pericardial diseases of the european society of cardiology (ESC)Endorsed by: the european association for cardio-thoracic surgery (EACTS). Eur Heart J.

[13] Nidorf S, Eikelboom J, Budgeon C, Thompson P (2013). Low-dose colchicine for secondary prevention of cardiovascular disease. J Am Coll Cardiol.

[14] Nidorf SM, Fiolet ATL, Mosterd A, Eikelboom JW, Schut A, Opstal TSJ, The SHK, Xu XF, Ireland MA, Lenderink T (2020). Colchicine in patients with chronic coronary disease. N Engl J Med.

[15] Tong D, Bloom J, Quinn S, Nasis A, Hiew C, Roberts-Thomson P, Adams H, Sriamareswaran R, Htun N, Wilson W (2021). Colchicine in patients with acute coronary syndrome: two-year follow-up of the Australian COPS randomized clinical trial. Circulation.

[16] Robertson S, Martínez G, Payet C, Barraclough J, Celermajer D, Bursill C, Patel S (2016). Colchicine therapy in acute coronary syndrome patients acts on caspase-1 to suppress NLRP3 inflammasome monocyte activation. Clin Sci.

[17] Yang M, Lv H, Liu Q, Zhang L, Zhang R, Huang X, Wang X, Han B, Hou S, Liu D (2020). Colchicine alleviates cholesterol crystal-induced endothelial cell pyroptosis through activating AMPK/SIRT1 pathway. Oxid Med Cell Longev.

[18] Cirillo P, Conte S, Pellegrino G, Barra G, De Palma R, Sugraliyev A, Golino P, Cimmino G (2022). Effects of colchicine on tissue factor in oxLDL-activated T-lymphocytes. J Thromb Thrombolysis.

[19] Cimmino G, Conte S, Morello A, Pellegrino G, Marra L, Calì G, Golino P, Cirillo P (2021). Colchicine inhibits the prothrombotic effects of oxLDL in human endothelial cells. Vascul Pharmacol.

[20] Tong D, Wilson A, Layland J (2016). Colchicine in cardiovascular disease: an ancient drug with modern tricks. Heart.

[21] Papageorgiou N, Briasoulis A, Lazaros G, Imazio M, Tousoulis D (2017). Colchicine for prevention and treatment of cardiac diseases: a meta-analysis. Cardiovasc Ther.

[22] Rosenblum D, Joshi N, Tao W, Karp J, Peer D (2018). Progress and challenges towards targeted delivery of cancer therapeutics. Nat Commun.

[23] Chen W, Glackin C, Horwitz M, Zink J (2019). Nanomachines and other caps on mesoporous silica nanoparticles for drug delivery. Acc Chem Res.

[24] Abdul Rahim R, Jayusman P, Muhammad N, Ahmad F, Mokhtar N, Naina Mohamed I, Mohamed N, Shuid A (2019). Recent advances in nanoencapsulation systems using PLGA of bioactive phenolics for protection against chronic diseases. Int J Environ Res Public Health.

[25] Chen J, Zhang X, Millican R, Sherwood J, Martin S, Jo H, Yoon YS, Brott BC, Jun HW (2021). Recent advances in nanomaterials for therapy and diagnosis for atherosclerosis. Adv Drug Deliv Rev.

[26] Suk J, Xu Q, Kim N, Hanes J, Ensign L (2016). PEGylation as a strategy for improving nanoparticle-based drug and gene delivery. Adv Drug Deliv Rev.

[27] Park J, Ryu S, Jung I, Lee Y, Kang K, Lee M, Lee M, Sonn S, Lee J, Lee H (2013). Evaluation of VCAM-1 antibodies as therapeutic agent for atherosclerosis in apolipoprotein E-deficient mice. Atherosclerosis.

[28] Zhou J, Guo D, Zhang Y, Wu W, Ran H, Wang Z (2014). Construction and evaluation of Fe_3_O_4_-based PLGA nanoparticles carrying rtPA used in the detection of thrombosis and in targeted thrombolysis. ACS Appl Mater Interfaces.

[29] Zhang W, Wang J, Xie Z, Zou H, Chen Q, Xu L, Hu L, Fang N, Xu J, Zhou J (2022). Antithrombotic therapy by regulating the ROS-mediated thrombosis microenvironment and specific nonpharmaceutical thrombolysis using prussian blue nanodroplets. Small.

[30] Zhong Y, Zhang Y, Xu J, Zhou J, Liu J, Ye M, Zhang L, Qiao B, Wang ZG, Ran HT (2019). Low-intensity focused ultrasound-responsive phase-transitional nanoparticles for thrombolysis without vascular damage: a synergistic nonpharmaceutical strategy. ACS Nano.

[31] Wang Y, Zhang K, Li T, Maruf A, Qin X, Luo L, Zhong Y, Qiu J, McGinty S, Pontrelli G (2021). Macrophage membrane functionalized biomimetic nanoparticles for targeted anti-atherosclerosis applications. Theranostics.

[32] Li Y, Che J, Chang L, Guo M, Bao X, Mu D, Sun X, Zhang X, Lu W, Xie J (2022). CD47- and Integrin α4/β1-comodified-macrophage-membrane-coated nanoparticles enable delivery of colchicine to atherosclerotic plaque. Adv Healthcare Mater.

[33] Meyer-Lindemann U, Mauersberger C, Schmidt AC, Moggio A, Hinterdobler J, Li X, Khangholi D, Hettwer J, Grasser C, Dutsch A (2022). Colchicine impacts leukocyte trafficking in atherosclerosis and reduces vascular inflammation. Front Immunol.

[34] Vaidya K, Arnott C, Martínez GJ, Ng B, McCormack S, Sullivan DR, Celermajer DS, Patel S (2018). Colchicine therapy and plaque stabilization in patients with acute coronary syndrome. JACC Cardiovascular Imaging.

[35] Zhang S, Liu Y, Cao Y, Zhang S, Sun J, Wang Y, Song S, Zhang H (2022). Targeting the microenvironment of vulnerable atherosclerotic plaques: an emerging diagnosis and therapy strategy for atherosclerosis. Adv Mater.

[36] Yurdagul A, Doran A, Cai B, Fredman G, Tabas I (2017). Mechanisms and consequences of defective efferocytosis in atherosclerosis. Front Cardiovas Med.

[37] Tabas I, Lichtman A (2017). Monocyte-macrophages and T cells in atherosclerosis. Immunity.

[38] Lemprière S (2020). NLRP3 inflammasome activity as biomarker for primary progressive multiple sclerosis. Nat Rev Neurol.

[39] Ising C, Venegas C, Zhang S, Scheiblich H, Schmidt S, Vieira-Saecker A, Schwartz S, Albasset S, McManus R, Tejera D (2019). NLRP3 inflammasome activation drives tau pathology. Nature.

[40] Deftereos SG, Beerkens FJ, Shah B, Giannopoulos G, Vrachatis DA, Giotaki SG, Siasos G, Nicolas J, Arnott C, Patel S (2022). Colchicine in cardiovascular disease: in-depth review. Circulation.

[41] Harrison S, Lane D, Lip G (2020). Reducing risk of adverse cardiovascular and renal outcomes for patients with atrial fibrillation and type 2 diabetes. Eur J Heart Fail.

[42] Nguyen LTH, Muktabar A, Tang J, Dravid VP, Thaxton CS, Venkatraman S, Ng KW (2017). Engineered nanoparticles for the detection, treatment and prevention of atherosclerosis: how close are we?. Drug Discovery Today.

[43] Kim YS, Park JS, Park M, Ko MY, Yi SW, Yoon JA, Yang S, Shim SH, Park K-H, Song H (2018). PLGA nanoparticles with multiple modes are a biologically safe nanocarrier for mammalian development and their offspring. Biomaterials.

[44] Zhang Y, Dong Y, Fu H, Huang H, Wu Z, Zhao M, Yang X, Guo Q, Duan Y, Sun Y (2021). Multifunctional tumor-targeted PLGA nanoparticles delivering Pt(IV)/siBIRC5 for US/MRI imaging and overcoming ovarian cancer resistance. Biomaterials.

[45] Jiang G, Huang Z, Yuan Y, Tao K, Feng W (2021). Intracellular delivery of anti-BCR/ABL antibody by PLGA nanoparticles suppresses the oncogenesis of chronic myeloid leukemia cells. J Hematol Oncol.

[46] Pan Z, Ding J (2012). Poly(lactide-co-glycolide) porous scaffolds for tissue engineering and regenerative medicine. Interface focus.

[47] Sadeghzadeh F, Ziaratnia AS, Homayouni Tabrizi M, Torshizi GH, Alhajamee M, Khademi D (2023). Nanofabrication of PLGA-PEG-chitosan-folic acid systems for delivery of colchicine to HT-29 cancer cells. J Biomater Sci Polym Ed.

[48] Distasio N, Dierick F, Ebrahimian T, Tabrizian M, Lehoux S (2022). Design and development of branched Poly(ss-aminoester) nanoparticles for Interleukin-10 gene delivery in a mouse model of atherosclerosis. Acta Biomater.

[49] Jia X, Bai X, Yang X, Wang L, Lu Y, Zhu L, Zhao Y, Cheng W, Shu M, Mei Q (2022). VCAM-1-binding peptide targeted cationic liposomes containing NLRP3 siRNA to modulate LDL transcytosis as a novel therapy for experimental atherosclerosis. Metabolism Clin Exp.

[50] Dosta P, Tamargo I, Ramos V, Kumar S, Kang DW, Borros S, Jo H (2021). Delivery of Anti-microRNA-712 to Inflamed Endothelial Cells Using Poly(beta-amino ester) Nanoparticles Conjugated with VCAM-1 Targeting Peptide. Adv Healthcare Mater.

[51] Zhang FS, He QZ, Qin CH, Little PJ, Weng JP, Xu SW (2022). Therapeutic potential of colchicine in cardiovascular medicine: a pharmacological review. Acta Pharmacol Sin.

[52] Li Y, Zhang Y, Lu J, Yin Y, Xie J, Xu B (2021). Anti-inflammatory mechanisms and research progress of colchicine in atherosclerotic therapy. J Cell Mol Med.

[53] Durpès M, Morin C, Paquin-Veillet J, Beland R, Paré M, Guimond M, Rekhter M, King G, Geraldes P (2015). PKC-β activation inhibits IL-18-binding protein causing endothelial dysfunction and diabetic atherosclerosis. Cardiovasc Res.

[54] Schwarz N, Fernando S, Chen YC, Salagaras T, Rao SR, Liyanage S, Williamson AE, Toledo-Flores D, Dimasi C, Sargeant TJ (2023). Colchicine exerts anti-atherosclerotic and -plaque-stabilizing effects targeting foam cell formation. FASEB J.

[55] Thompson PL, Nidorf SM (2018). Colchicine: an affordable anti-inflammatory agent for atherosclerosis. Curr Opin Lipidol.

